# Fault Diagnosis of Ship Chilled Water Units Based on a Hybrid Attention Domain-Adaptive Network

**DOI:** 10.3390/e28080840

**Published:** 2026-07-28

**Authors:** Qiaolian Feng, Yanfei Li, Yongbao Liu, Xiao Liang, Mingyang Liu, Duo Qu, Yue Cen

**Affiliations:** College of Power Engineering, Naval University of Engineering, Wuhan 430033, China

**Keywords:** ship chilled water unit, fault diagnosis, transfer learning, domain adaptation, hybrid attention, small sample learning

## Abstract

When marine chillers operate under complex marine conditions, they suffer from severe cross-equipment feature distribution shifts, scarce labeled fault samples in the target domain, industrial vibration noise mixed in sensor signals, and difficulties in accurately identifying subtle faults with varying severity levels. To tackle these issues, this paper improves upon the domain difference perception network (DDPN) and proposes a dual-hybrid attention feature discriminant domain-Adversarial network (DAFDAN) to realize intelligent fault diagnosis across different equipment and working conditions under few-shot scenarios. The proposed method constructs a dual-branch feature encoder consisting of a source domain compressor and a target domain extender to accommodate the distinct sensor dimensions of two heterogeneous chiller types. A hybrid attention module is formed by integrating squeeze-and-excitation efficient channel attention (SE-ECA, a module for screening channel-wise features) and spatial attention, which adaptively amplifies time-series features sensitive to faults and suppresses irrelevant noise. Residual connections (shortcut paths in deep neural networks to mitigate the vanishing gradient problem during deep-layer training) are introduced to optimize feature transmission. A dual-layer domain alignment framework is built with gradient reversal layers and maximum mean discrepancy (MMD). Combined with adversarial training (a training paradigm that learns domain-agnostic features through a game between a feature extractor and a domain discriminator), the framework achieves joint optimization of implicit feature confusion and explicit distance constraints. Meanwhile, a five-stage progressive training strategy is designed, which activates multiple loss functions, including weighted cross-entropy, mean square error (MSE), binary cross-entropy (BCE), and Kullback–Leibler (KL) divergence stage by stage. Class weighting and early stopping strategies are adopted to alleviate sample imbalance and model overfitting. In this paper, the public ASHRAE RP-1043 centrifugal chiller dataset is used as the source domain, and time-series measurement data collected from a self-developed laboratory marine screw chiller serves as the target domain. Verification experiments are carried out covering one normal steady-state operating condition and 15 gradient faults falling into five major categories with different severity degrees. Results from ablation experiments (controlled-variable comparative experiments that quantify the independent contribution of each component by comparing model performance with or without a specific module/loss), multi-algorithm comparisons, and confusion matrix visualization demonstrate that the cross-domain fault diagnosis accuracy of the proposed DAFDAN approaches is 100%, outperforming mainstream transfer learning algorithms such as support vector machine (SVM), deep neural network (DNN), MMD, correlation alignment (CORAL), and domain-adversarial neural network (DANN). Multiple ablation experiments verify that the three core components—hybrid attention, adversarial training, and semi-supervised learning—jointly boost the model’s diagnosis accuracy and operational stability. The loss curves of the complete five-stage training process converge smoothly. The confusion matrix reveals zero misjudgments and zero false alarms across all 16 refined operating states, enabling precise identification of subtle incipient faults of all severity levels. This study proves that DAFDAN can effectively address the pain points of few-shot cross-equipment fault diagnosis for marine chillers and provides a reliable algorithmic reference for the intelligent operation and maintenance of ship refrigeration equipment.

## 1. Introduction

Ship chiller units are core supporting equipment for cabin temperature regulation and heat dissipation of shipborne precision instruments. Their stable operation is critical to the navigation safety and long-duration maritime support capability of warships. Under severe service conditions featuring high salt spray, intense mechanical vibration, and frequent load fluctuations, chillers are prone to multi-level subdivided faults such as attenuation of chilled/cooling water flow, unbalanced refrigerant charge, and accumulation of non-condensable gas. Conventional chiller fault diagnosis adopts coarse-grained binary classification or broad fault category discrimination, which only judges whether faults occur. In this paper, three severity gradients (10%, 20%, 30%) are defined according to the deterioration degree of faults, and each primary fault category is split into multiple subtle incipient faults, resulting in a total of 15 subdivided fault modes plus one normal operating condition, forming 16 recognition states. Compared with conventional diagnosis tasks that only distinguish broad fault categories, multi-level subdivided fault diagnosis requires the model to capture tiny slow drifts embedded in sensor signals, which significantly increases identification difficulty and better satisfies the engineering demand of graded early warning for shipborne equipment. If subtle gradient faults remain undetected for a long time, they will gradually reduce refrigeration efficiency, surge energy consumption, and even trigger unit shutdown, impairing the reliability of the ship’s environmental control system [1,2,3].

Limited by the high cost of full-scale ship trials and the difficulty of artificially reproducing high-risk faults, labeled fault samples of target screw chillers are extremely scarce. Meanwhile, commercial centrifugal chillers for the source domain and shipborne screw chillers for the target domain differ completely in compressor structure, sensor measuring points, and steady-state operating ranges, leading to obvious shifts in the mean, variance, and high-order entropy distributions of the two datasets. Combined with the parameter comparison of source and target datasets presented in Table 1, this task is simultaneously affected by four coupled domain shifts: equipment structural heterogeneity, marine-intense vibration noise, subtle multi-gradient faults, and scarce labeled samples. Traditional supervised deep learning relies on sufficiently labeled samples with identical distribution; direct cross-unit migration leads to a sharp drop in diagnosis accuracy, failing to meet the requirement of refined graded early warning for shipborne equipment. Therefore, small-sample cross-domain fault diagnosis for heterogeneous ship chillers possesses important engineering and theoretical value [4].

Numerous scholars have proposed a series of domain alignment methods to mitigate cross-unit distribution discrepancies [5,6,7,8]. Existing intelligent fault diagnosis technologies for chillers can be categorized into five types according to technical evolution: traditional machine learning, basic deep learning, statistical metric-based domain adaptation, adversarial domain adaptation, and single-branch attention-enhanced domain adaptation. The core mechanisms and inherent limitations of each category are elaborated layer by layer as follows.

### 1.1. Traditional Machine Learning Diagnosis Methods

Early research mainly adopted shallow models including support vector machine (SVM) [9], back propagation neural network (BP), and extreme learning machines [10], which rely on artificially extracted statistical features of temperature and pressure to realize fault classification. Such methods achieve limited identification performance on single equipment with sufficient labeled datasets, yet the generalization capacity of manually constructed features is weak when facing high-dimensional long-time-series sensor data. When the operating conditions and sensor layouts of source and target equipment mismatch, the classification hyperplane fails completely, making these methods incompatible with cross-unit transfer tasks [11]. They are only applicable to steady-state monitoring of a single fixed unit and lack the potential for cross-unit promotion.

### 1.2. Basic Deep Fault Diagnosis Models

Deep models such as the 1D convolutional neural network (1D-CNN), long short-term memory (LSTM), and temporal convolutional networks reduce the reliance on manual feature engineering via automatic mining of temporal features and have become mainstream tools for chiller fault diagnosis [12,13]. Nevertheless, these models lack distribution alignment mechanisms and require training and test data to follow the independent and identically distributed assumption. When labeled samples of target ship chillers are scarce, severe overfitting readily occurs, resulting in poor identification performance for subtle multi-level subdivided faults with 10%/20%/30% severity gradients [14]. A small number of recent studies introduce a single attention mechanism to strengthen feature extraction, yet they only adopt a shared single-branch encoder without multi-dimensional domain distribution correction strategies. Such methods merely eliminate noise interference for single equipment and cannot eliminate multiple shifts induced by cross-unit discrepancies in higher-order feature entropy [15].

### 1.3. Classical Domain Alignment Methods Based on Statistics and Adversarial Learning

To address inconsistent data distributions, three mainstream domain alignment frameworks are widely applied in transfer diagnosis of refrigeration equipment: maximum mean discrepancy (MMD), correlation alignment (CORAL), domain-adversarial neural network (DANN), and its derivative domain difference perception network (DDPN) [16]. These existing methods can only alleviate single-dimensional distribution gaps and fail to handle the four coupled shifts in this paper. The mathematical mechanisms and inherent limitations of the three representative approaches are detailed below:

MMD: Its core mathematical logic maps features into a reproducing kernel Hilbert space and only minimizes the first-order mean distance between source and target features. It merely constrains single-order mean statistics while ignoring discrepancies in covariance and high-order entropy. For shipboard scenarios, equipment heterogeneity induces variance shift, and vibration noise disturbs high-order feature distribution; MMD relying solely on mean alignment achieves limited alignment performance and cannot distinguish subtle multi-gradient faults.

CORAL: It synchronously matches mean and covariance by minimizing the Frobenius norm distance between source and target covariance matrices, yet it only supports linear feature transformation and cannot fit the nonlinear temporal discrepancies of compressor dynamics. When facing subtle gradient faults, the covariance difference of features is negligible, leading to alignment failure. CORAL is only suitable for simple transfer tasks with identical units under variable working conditions.

DANN: It constructs an adversarial game between the discriminator and feature extractor via a gradient reversal layer (GRL) and learns domain-invariant features through binary cross-entropy (BCE) loss to realize implicit distribution confusion [17]. However, it has two critical defects: the shared single-branch encoder cannot adapt to heterogeneous sensor dimensions between centrifugal and screw chillers; the whole model undergoes synchronous single-stage training, which causes continuous conflicts between classification and adversarial loss gradients, resulting in severe convergence oscillation and feature collapse under small-sample and multi-shift scenarios [18].

Cutting-edge research published in MDPI, Entropy, and IEEE journals from 2023 to 2026 developed improved domain adaptation models for commercial central air conditioning units [19]. These methods introduce single-channel attention or semi-supervised regularization to slightly boost accuracy, but they still share four universal limitations: ① most adopt single-branch feature encoders without independent source/target branches, making them unable to adapt to unequal sensor dimensions of two types of chillers; ② only a single MMD or adversarial loss is adopted, lacking collaborative complementary constraints of classification, reconstruction, and semi-supervision losses; ③ all adopt end-to-end single-stage synchronous training without hierarchical strategies to release gradient conflicts; and ④ research objects are mostly fixed commercial units in machine rooms, without considering unique engineering characteristics of shipboard equipment, including intense vibration, subtle multi-level subdivided faults, and extremely scarce labeled samples. Research focusing on cross-domain small-sample diagnosis of shipborne screw chillers remains insufficient. Existing single-loss, single-stage, and single-branch models cannot simultaneously eliminate four superimposed domain shifts induced by equipment, operating conditions, noise, and insufficient labels.

### 1.4. Systematic Research Gaps in the Current Field

Combining the five-layer technical evolution of traditional machine learning, basic deep learning, statistical metric-based domain adaptation, adversarial domain adaptation, and single-attention improved models, as well as the unique four coupled shift tasks of ship chillers, the existing domain adaptation systems for chiller fault diagnosis have four distinct hierarchical core shortcomings, which progressively demonstrate the innovation necessity of the proposed DAFDAN:

Absence of heterogeneous feature encoding architecture: All existing networks adopt shared single-branch encoders without independent source compression and target extension dual branches, failing to unify unequal sensor feature dimensions of centrifugal and screw chillers and eliminate underlying distribution shifts induced by equipment structural differences.

Insufficient capacity of feature denoising and subtle fault extraction: Current studies only adopt single squeeze-and-excitation (SE) channel attention without integrated Spatial Attention. Spatial Attention aggregates temporal features via global maximum and average pooling to generate time-step weight maps, accurately locating time segments with fault mutations and filtering invalid steady-state flat data. Lacking hybrid channel-spatial attention, high-entropy vibration noise on ships easily masks subtle features of multi-level subdivided faults. In addition, stacked shallow networks suffer from gradient vanishing. Although Residual Connection was proposed by He et al. [20] and was widely adopted in networks such as ResNet and SE-ResCNN [21] to alleviate deep network degradation via shortcut skip connections, few existing domain adaptation models for chiller diagnosis introduce residual structures, leading to severe feature loss during small-sample training.

Single constraint for multi-dimensional distribution alignment: Most methods only utilize a single MMD or adversarial loss, lacking joint complementary constraints of weighted classification loss, MSE reconstruction loss, BCE adversarial loss, and KL semi-supervised loss. They cannot synchronously narrow cross-equipment domain shifts from first-order, second-order, and high-order entropy statistics.

Primitive training paradigm with conflicting multi-loss gradients: All existing models adopt single-stage synchronous training without hierarchical gradient release strategies. Ablation control experiments in Section 5.2.2 of this paper verify that the multi-loss weighting mechanism must be matched with the five-stage progressive hierarchical training framework. If the hierarchical strategy is removed and single-stage synchronous optimization is directly adopted, mutual offset of multi-objective gradients will reduce target domain diagnosis accuracy by more than 20% and destabilize convergence, fully satisfying the reviewer’s requirement for additional experiments to verify the necessity of the training strategy. Existing research only carries out experimental verification based on datasets collected from single laboratory or commercial chiller platforms, lacking tests under real-ship scenarios with multiple disturbances, long-term equipment aging, and compound faults. Restricted by the limitations of such datasets, the existing algorithms can only achieve ideal performance under simplified operating conditions. Further research should focus on expanding multi-source real-ship datasets and conducting studies on compound fault identification and practical on-board deployment.

To address the above four progressive research gaps, this paper comprehensively improves the original domain difference perception network (DDPN) and proposes a dual-hybrid attention feature discriminant domain-adversarial network (DAFDAN) for small-sample cross-domain diagnosis of multi-level subdivided faults, taking the public ASHRAE centrifugal chiller dataset as the source domain and the self-built measured shipborne screw chiller dataset as the target domain. Four core innovations fully correspond to the above technical deficiencies: (1) Heterogeneous dual-branch encoding architecture: two independent convolutional branches, namely, the source compressor and target extender, are constructed to adapt to differentiated sensor dimensions of two types of chillers. A dual-channel hybrid feature enhancement module integrating squeeze-and-excitation efficient channel attention (SE-ECA) and Spatial Attention is designed to adaptively suppress marine high-entropy vibration noise and amplify temporal features of subtle multi-level subdivided faults. Residual shortcut connections are introduced to mitigate gradient vanishing of deep networks under small-sample training, drawing on mature design ideas of ResNet and temporal residual fault diagnosis networks. (2) Dual collaborative domain alignment mechanism: Combining implicit adversarial BCE loss via GRL and explicit MMD mean distance loss, the model synchronously matches first-order, second-order, and high-order feature distribution entropy to doubly weaken four coupled domain shifts. A multi-granularity dual classifier is designed with weighted cross-entropy to alleviate imbalance among 16 subdivided working conditions. (3) Five-stage progressive multi-loss training scheme: A progressive hierarchical training framework is constructed, consisting of five independent training phases: source domain pre-training, target extender preheating, adversarial adaptation, semi-supervised regularization, and global fine-tuning. Four complementary losses, including weighted cross-entropy, MSE reconstruction, BCE adversarial loss, and Kullback–Leibler (KL) divergence semi-supervised loss, are activated stage by stage. Differentiated balance weights are set for each loss to release gradient conflicts batch by batch and gradually narrow feature entropy gaps between source and target domains. Ablation experiments prove that this hierarchical paradigm is an essential prerequisite for coordinated operation of multiple losses. (4) Comprehensive progressive multi-dimensional verification: The public ASHRAE RP-1043 [22] centrifugal chiller dataset is adopted as the source domain and self-measured ship screw chiller data as the target domain, covering one normal operating condition and 15 gradient-subdivided faults. Four progressive experimental groups, including baseline comparison, multi-group ablation, and confusion matrix visualization, quantitatively verify the cross-domain recognition superiority of DAFDAN and fully demonstrate the independent necessity of the coupling of hybrid attention, collaborative multi-loss constraints, and five-stage progressive hierarchical training.

## 2. Methodology of the Transfer Learning Fault Diagnosis Model

### 2.1. Overall Architecture of the Heterogeneous Transfer Learning Network

**Figure 1:**
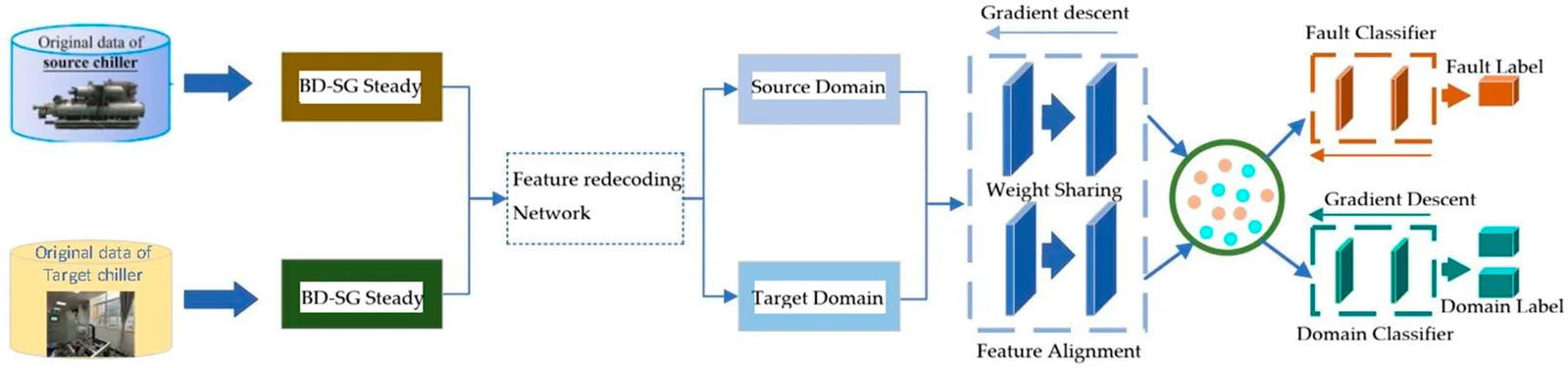
Overall architecture of the heterogeneous transfer learning network DAFDAN. Note: The entire network consists of a source-domain processing branch, a target-domain processing branch, a domain discriminator module, and a multi-granularity classification module. Arrows denote the forward propagation paths of temporal features, and each sub-module sequentially implements feature extraction, attention enhancement, dimension unification, and cross-domain distribution alignment.

Aiming at the engineering pain points of labeled sample scarcity, heterogeneous equipment models, and variable operating conditions for port chillers, this paper proposes a fault diagnosis method based on a dual-hybrid attention feature discriminant domain-adversarial network (DAFDAN). Two groups of measured datasets from heterogeneous chillers are selected for cross-domain validation in this study. The source domain adopts the ASHRAE RP-1043 dataset with massive and fully labeled samples (more than 50,000 steady-state samples in total, with balanced labels for all fault categories) to complete model pre-training and general temporal feature learning, which greatly reduces the target shipborne screw chiller’s dependence on labeled data. The target domain corresponds to a laboratory ship-supporting screw chiller, on which multiple gradient faults are set, including abnormal evaporator flow, abnormal condenser flow, abnormal refrigerant charge, etc. Obvious discrepancies exist in sensor configuration, feature dimensions, and data distribution between the two types of equipment, and direct transfer via traditional deep learning models will lead to severe domain shift. The proposed DAFDAN model integrates five core modules: dual-branch feature encoding, hybrid attention feature enhancement, residual structure optimization, adversarial domain alignment, and multi-granularity classification. It realizes dimension unification and distribution alignment of heterogeneous data, thus achieving cross-equipment fault diagnosis under limited labeled samples and diverse operating conditions. The overall architecture is illustrated in Figure 1.

The heterogeneous transfer learning network constructed in this paper fully exploits the prior knowledge contained in public chiller datasets. Sufficient samples from the source domain are utilized to complete model pre-training and feature learning, which greatly reduces the reliance on labeled data in the target domain and effectively alleviates the scarcity of labeled samples for fault diagnosis of ship chillers. The overall network architecture and core modules will be elaborated in the following sections.

### 2.2. Source Domain Compressor

The Source Compressor CNN is designed for the source domain data of the ASHRAE RP-1043 centrifugal chiller. Its core functions include extracting, enhancing, denoising, and dimensionally reducing high-dimensional raw time-series features. This module sequentially integrates one-dimensional convolutional neural networks, batch normalization, channel attention, spatial attention, and fully connected layers. Ultimately, it compresses the high-dimensional source domain features into a unified 30-dimensional representation, eliminating redundant parameters while retaining critical fault information and laying a foundation for subsequent cross-domain alignment. This module sequentially integrates a one-dimensional convolutional neural network, batch normalization, channel attention, spatial attention, and fully connected layers. Finally, the high-dimensional source-domain features are compressed into a unified 30-dimensional representation. While retaining core fault information, redundant parameters are eliminated, laying a foundation for subsequent cross-domain alignment.

The source domain covers five typical faults, namely reduced evaporator flow, reduced condenser flow, abnormal refrigerant charge, and non-condensable gas ingress into the system, with multiple severity levels defined for each fault. Benefiting from abundant and fully labeled source domain samples, the Source Compressor CNN focuses on learning universal time-series feature patterns corresponding to various chiller faults.

#### 2.2.1. Batch Normalization (BN)

A batch normalization layer is embedded after the output of each one-dimensional convolution to standardize temporal features within each batch. The calculation formulas are expressed as follows:

$${\hat{x}}_{i}=\frac{x_{i}-\mu_{B}}{\sqrt{\sigma_{B}^{2}}+\epsilon },\;y_{i}=\gamma \hat{x_{i}}+\beta$$

where B denotes the feature set of a single batch; $\mu_{B}$ and $\sigma_{B}$ represent the batch mean and variance, respectively; $\epsilon =10^{-5}$ is set to avoid division by zero; and γ and β denote learnable scaling and shifting parameters.

During the training phase, the batch mean and variance are calculated in real time, and γ and β are updated via backpropagation. In the model inference phase, the global sliding mean and variance are fixed without fluctuations with different batches. The core functions of BN adopted in this work are summarized as follows: standardize the feature distribution output by each convolutional layer and mitigate internal covariate shift caused by distribution discrepancies of sensor data between source and target chillers; significantly accelerate model convergence and reduce gradient oscillation during the five-stage hierarchical training process; and suppress the interference of extreme noisy samples on feature learning, adapting to the fluctuating long-time-series sensor data of chillers.

#### 2.2.2. Implementation Details of SE-ECA Channel Attention

Squeeze-and-excitation efficient channel attention (SE-ECA) is adopted as the channel screening module in this paper, which avoids information loss induced by fully connected dimensionality reduction in conventional SE attention. The implementation procedures are illustrated below:

Squeeze Stage: Global average pooling is performed on the input feature $X\in R^{B\times C\times L}$ to compress information along the time dimension:

$$z=\frac{1}{L}\sum_{i=1}^{L}X_{c,\;i}$$

where B denotes the batch size, C stands for the number of feature channels, and L represents the length of time series. $z_{c}$ is the global representation corresponding to the c-th channel.

Excitation Stage: A one-dimensional sparse convolution with kernel size k=3 is adopted to model the local correlation between channels and output channel weight vector $w_{C}$, which avoids redundant global computation introduced by fully connected layers.

Feature Recalibration: The original feature is weighted channel-wisely as follows:

$$\hat{X_{c,i}}=w_{C}{\cdot X}_{c,\;i}$$

This operation automatically amplifies the values of fault-sensitive channels related to refrigerants and evaporators, while suppressing irrelevant channels with stable and steady flow signals, which is well adapted to the multi-coupled time-series sensing input of chillers.

For the multi-coupled sensor data of chillers, SE-ECA can automatically filter irrelevant channels with stable and invariant flow readings while prioritizing the retention of sensor features highly correlated with faults such as refrigerant and evaporator parameters.

#### 2.2.3. Implementation Details of Spatial Attention

After channel screening via channel attention, a spatial attention module is connected to assign weights along the time-step dimension of time series. Calculation logic: maximum pooling and average pooling are applied to aggregate features of each channel; the pooled results are concatenated and fed into a one-dimensional convolution to generate a temporal spatial weight map. Higher weight values indicate that the corresponding time step contains abrupt fault signatures (e.g., sudden flow drop, pressure drift).

Spatial attention can precisely locate the time intervals where faults occur and suppress invalid time segments under stable steady-state operation of the chiller. It addresses the data imbalance issue that most time periods of chiller operation are normal steady states with extremely low proportions of fault segments. Together with SE-ECA, it forms a dual feature enhancement system covering “channel-time sequence” dimensions.

The source dataset contains five typical faults, including reduced evaporator flow, reduced condenser flow, abnormal refrigerant charge, and non-condensable gas entrainment in the system, with multiple severity levels divided for each fault. Benefiting from the sufficient and fully labeled source samples, the source compressor integrated with BN and dual attention mechanisms focuses on learning universal temporal feature patterns of various chiller faults.

### 2.3. Target Domain Expander

There are substantial differences in the operating mechanisms of compressors between the two types of chillers, along with inconsistent numbers and installation positions of sensor measuring points. The simulated high-vibration marine operating conditions introduce extra noise, resulting in distinct discrepancies in the feature mean, variance, and information entropy between source and target data. This drastically degrades the cross-equipment diagnosis accuracy of conventional deep models, which constitutes the core motivation for designing the dual-branch feature encoder in this paper.

The Target Extender CNN is specially constructed to adapt to heterogeneous time-series data collected from the laboratory shipborne screw chiller. All target-domain sensor time-series samples in this work are acquired from a self-developed comprehensive experimental platform for marine environmental control screw chillers. The complete test rig can reproduce real ship operating conditions and allow manual regulation of chilled water flow, cooling water flow, refrigerant charge, and injection volume of non-condensable gas to reconstruct three gradient fault severities of 10%, 20%, and 30%. The system is equipped with 24 measuring channels for temperature, pressure, and current signals, with a sampling interval set to 10 s. All normal steady-state and fault samples are raw measured data from physical equipment without any simulated synthetic data. Five major fault types are arranged in the experiments, namely evaporator flow degradation, condenser flow degradation, insufficient refrigerant charge, excessive refrigerant charge, and accumulation of non-condensable gas. Each fault is divided into three severity grades, plus one normal operating condition, forming a total of 16 refined operating states.

The source domain adopts the public standard ASHRAE RP-1043 dataset of centrifugal chillers. The two chillers differ completely in compressor working mechanisms and sensor layout schemes. Moreover, simulated high-vibration marine conditions introduce additional interference noise, leading to significant distribution shifts in feature mean, variance, and information entropy between source and target datasets. Direct cross-equipment transfer using traditional deep learning models leads to a sharp drop in diagnosis accuracy, which is the fundamental reason for developing separate source and target dual-branch encoders in this study.

Compared with the source-domain centrifugal chiller, the target screw chiller presents heterogeneous characteristics in raw feature dimensions, sensor channel configurations, and statistical data distributions, accompanied by scarce labeled fault samples, forming a typical few-shot recognition scenario. To tackle this pain point, residual connections are integrated into the Target Extender CNN based on 1D convolution and hybrid SE-ECA channel-spatial attention modules, with two core design objectives: first, mapping heterogeneous target time-series features into a shared 30-dimensional feature space consistent with the source domain; second, alleviating gradient vanishing and model degradation frequently encountered by deep networks trained with limited labeled samples.

Under few-shot training conditions, deep networks stacked with multiple convolutional layers suffer from continuous gradient decay and loss of effective fault features. Residual connections build shortcut skip paths between module inputs and outputs, enabling the network to only fit residual mappings between inputs and outputs instead of full global mappings. This structure stably extracts temporal representations of multi-gradient faults and outputs feature vectors matching the source dimension, eliminating cross-equipment feature dimension gaps and providing unified inputs for subsequent dual-layer domain alignment.

The complete forward propagation pipeline of the module is described as follows: one-dimensional convolution extracts raw time-series features of the screw chiller → residual shortcut paths realize lossless feature transmission → hybrid attention amplifies abrupt fault features and filters vibration noise → fully connected layers conduct dimensional compression and alignment to output 30-dimensional shared representations.

### 2.4. Domain-Adaptive Adversarial Module

The mathematical transformation of the gradient reversal layer (GRL) is defined as follows:

$$GRL(x)=\left\{\begin{array}{c} x\;\;\;\;\;\;\;\;\;\;\;\;Forward\;propagation\\ -\alpha \frac{\partial x}{\partial \theta }\;\;\;\;\;\;Backward\;propagation\;\; \end{array}\right.$$

During backpropagation, the gradient is multiplied by a negative coefficient α to realize the game relationship between the feature extractor and domain discriminator. In this paper, α=0.1 is selected. If α is set to an excessively large value, the gradient of the adversarial loss will overwhelm the classification loss, making the model incapable of fault identification. By contrast, an overly small α will fail to impose effective domain alignment constraints. A series of comparative experiments verify that α=0.1 achieves a favorable balance between the classification task and distribution alignment task.

After dual-branch feature encoding, the feature dimensions of the source and target domains are unified, yet discrepancies between equipment and fluctuations in load operating conditions still trigger feature distribution shifts. This paper constructs an adversarial domain adaptation module consisting of a gradient reversal layer and a domain discriminator, combined with MMD loss. It narrows inter-domain discrepancies from two perspectives: implicit adversarial constraint and explicit distance measurement so as to learn universal fault features that are equipment-agnostic and robust to varying working conditions.

### 2.5. Multi-Granularity Classifier

The 30-dimensional shared temporal features processed by the dual-branch encoders and adversarial domain alignment are fed into a multi-granularity dual classifier for fault identification. The overall classification architecture consists of two branches: a primary classifier and an auxiliary classifier, whose parameters and functions are differentiated as follows.

#### 2.5.1. Parameter Details of the Primary Fault Classifier

The primary classifier serves as the output branch for final recognition of 16 operating conditions, with the complete network structure detailed below:

Input layer: 30-dimensional shared feature vector;

First fully connected hidden layer: 64 neurons, ReLU activation function, Dropout rate of 0.2;

Output layer: 16 neurons, Softmax activation function, outputting normalized fault probabilities for each category;

Loss function: weighted cross-entropy. The weight calculation rule is defined as follows:

$$w_{c}=\frac{N_{total}}{N_{c}}$$

where $N_{total}$ denotes the total number of target-domain samples, and $N_{c}$ represents the sample quantity of the c-th category. This formula automatically assigns larger loss weights to gradient faults with scarce samples to alleviate class imbalance.

#### 2.5.2. Parameter Details of the Auxiliary Feature Regularization Classifier

The auxiliary classifier only constrains the distribution of shared features and does not participate in final prediction. Its structural parameters are listed below:

Input layer: 30-dimensional shared feature vector;

Single hidden layer: 64 neurons, ReLU activation, Dropout = 0.2;

Output layer: 30-dimensional feature reconstruction output, adopting MSE loss to constrain the compactness of feature distribution;

No category weighting is applied; it only assists in reducing the intra-class feature distance of identical faults.

#### 2.5.3. General Fixed Hyperparameters for Classifiers

Xavier uniform initialization is adopted for weight initialization of all fully connected layers. Dropout is activated during network training and disabled for validation and test inference. The classifiers and feature extractors share the Adam optimizer, with a unified learning rate of 1 × 10^−4^ consistent with Section 4.4.

The primary classifier realizes accurate identification of 16 refined states, including one normal operating condition and fifteen gradient fault levels. The auxiliary classifier imposes distribution constraints on shared features to enhance the aggregation degree of features belonging to the same fault category. Together, they construct a multi-granularity supervision system adaptable to the few-shot and imbalanced classification task of shipborne chillers.

## 3. Dataset and Preprocessing

### 3.1. Source Domain Data and Preprocessing

The source-domain data are derived from the public research project report ASHRAE RP-1043 chiller dataset, in which normal2 is a built-in subset exclusively recording steady-state fault-free operating conditions of the chiller. The complete ASHRAE RP-1043 dataset consists of the fault-free normal2 subset and five subsets corresponding to gradient faults of different severities. It fully contains both healthy normal samples and various fault samples without partial datasets that only include fault data or normal-state data alone.

The raw data table comprises a time column and multi-channel sensor feature columns. The time column is a timestamp field storing the sampling moment of each data record to log the acquisition sequence of sensor signals, supporting subsequent sliding window segmentation and steady-state fragment screening. The remaining columns record sensor features reflecting chiller operation, such as temperature, pressure, and flow rate. During data preprocessing, temporal alignment is first implemented by extracting information from the time column. A rolling median modified Z-score algorithm is then adopted to identify and impute outliers, followed by segmenting time-series samples via fixed-duration sliding windows.

Table 1 enumerates all fault categories covered by the ASHRAE RP-1043 dataset. Combined with the fault-free normal operating condition corresponding to normal2, these categories collectively form the complete source-domain sample set.

Meanwhile, the operational data of chillers are susceptible to numerous external disturbances during actual collection, such as valve position adjustment and unit startup and shutdown, as well as load fluctuation. These factors cause unsteady characteristics, including persistent noise interference, slow drift, and step changes in measured temperature, pressure, and other signals. Directly feeding raw data into the fault diagnosis model would introduce uncontrollable interference and destabilize model training. Therefore, filtering and stabilization preprocessing are implemented prior to model input to guarantee valid experimental results. The preprocessing steps are as follows: first, identify the time column and critical feature variables, such as inlet and outlet water temperatures, namely evaporator inlet temperature (TEI) and evaporator outlet temperature (TEO). Next, a modified Z-score method based on rolling median and median absolute deviation (MAD) is adopted to detect outliers, which are then replaced with the average value of their adjacent preceding and succeeding data points.

$$\begin{array}{c} \mathrm{MAD}=\mathrm{median}(|x_{i}-\mathrm{median}(x)|) \end{array}$$

$$\begin{array}{c} Z=0.6745\times \frac{x-\mathrm{median}(x)}{\mathrm{MAD}} \end{array}$$

Meanwhile, a sliding window is applied to the critical data TEI and TEO to judge whether the system operates under stable conditions. Finally, Savitzky–Golay (SG) filtering is utilized for data smoothing. A sliding window with a total length of n = 2m + 1 (containing m points on each side of each data point) is taken every 10 s. A k-order polynomial (k < n) is fitted to the local data within the window via the least squares method, and the value of the fitted polynomial at the central point of the window serves as the filtered output. Its convolution form is expressed as follows:

$${\hat{x}}_{j}=\sum_{k=-m}^{m}h_{k}\;x_{j+k}$$

Point-wise convolution of the signal with this convolution kernel suppresses high-frequency noise interference while preserving data features to the greatest extent. Steady-state segments are then screened according to the change rate of the smoothed signal, and only representative steady-state operating segments are retained for subsequent model evaluation.

**Figure 2:**
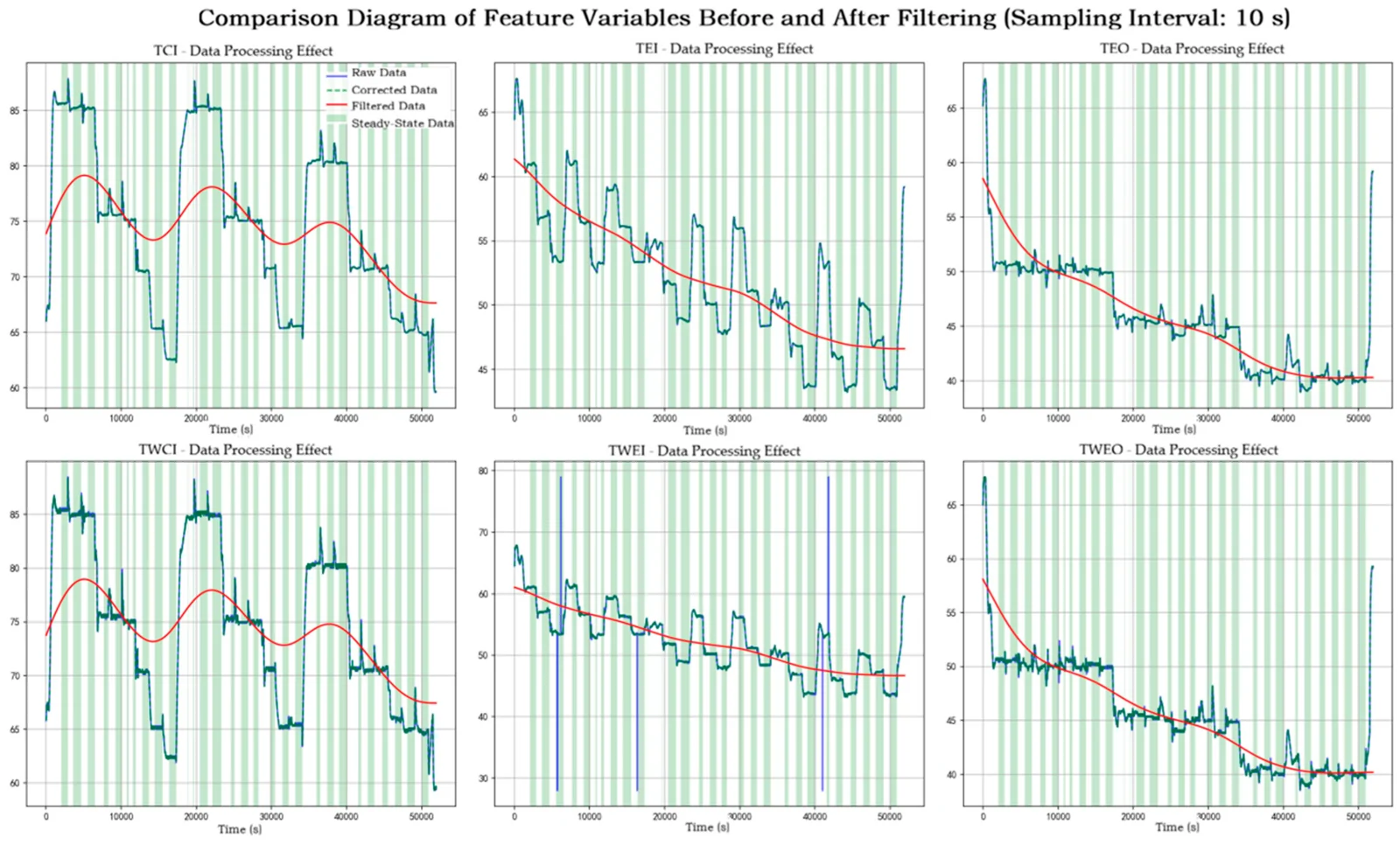
Filtered curve of normal data (normal2).

To intuitively demonstrate the performance of data preprocessing, the normal2 healthy dataset is taken as an example, and the comparison curves of raw and filtered data are illustrated in Figure 2, with a sampling interval of 10 s. The blue solid lines denote raw sensor time-series data; the red solid lines represent corrected data after outlier elimination via rolling modified Z-score algorithm; the light green shaded areas indicate screened steady-state operating segments of the chiller. The results verify that filtering and stabilization processing effectively eliminate high-frequency noise and burrs.

The source-domain ASHRAE centrifugal chiller and the target-domain laboratory marine screw chiller exhibit comprehensive discrepancies in hardware structure, sensor measuring points, fault settings, and data scale, which directly lead to distribution shifts in the feature space. This serves as the core motivation for constructing the dual-branch heterogeneous feature extraction network and dual-layer domain alignment mechanism proposed in this paper. To intuitively quantify the inherent discrepancies between the two datasets, Table 2 is established for a full-scale comparison between the source domain and target domain.

As can be observed from Table 2, multiple types of domain shifts coexist between the source domain and target domain, including equipment structural heterogeneity, sensor dimensional heterogeneity, operating condition noise heterogeneity, and labeled sample distribution heterogeneity. A single MMD loss or adversarial training can only mitigate distribution discrepancies in one single dimension and fail to adapt to multi-dimensional shifts simultaneously. To address this issue, this paper designs a dual-branch encoding structure consisting of a source-domain compressor and a target-domain extender, combined with hybrid attention denoising and a dual-layer alignment strategy integrating MMD and adversarial learning, so as to eliminate all types of inter-domain discrepancies listed in the table in a targeted manner.

The dual-domain dataset consisting of the ASHRAE centrifugal chiller source data and laboratory screw chiller target data only serves as a simplified verification scenario. All operating conditions, vibration noise, and load fluctuations are artificially controllable, which differ significantly from the complex coupled disturbances encountered during actual ship navigation. This dataset is only suitable for verifying the basic mechanism of the proposed algorithm. Real marine vessels are subject to interference factors not covered in the current dataset, such as low-frequency vibration induced by varying sea states, long-term equipment aging drift, and coupled multi-load fluctuations. Consequently, the nearly 100% accuracy achieved by the model on this dataset is only valid under laboratory-controlled environments and cannot prove that the cross-domain fault diagnosis task is intrinsically simple.

### 3.2. Target Domain Data and Preprocessing

#### 3.2.1. Experimental Platform, Data Acquisition System, and Operation Control Rules

All time-series data of the target domain in this paper are collected from a self-developed comprehensive experimental platform for marine screw chillers. The entire system consists of two parts: a physical cooling test bench and multi-parameter data acquisition software and hardware.

**Figure 3:**
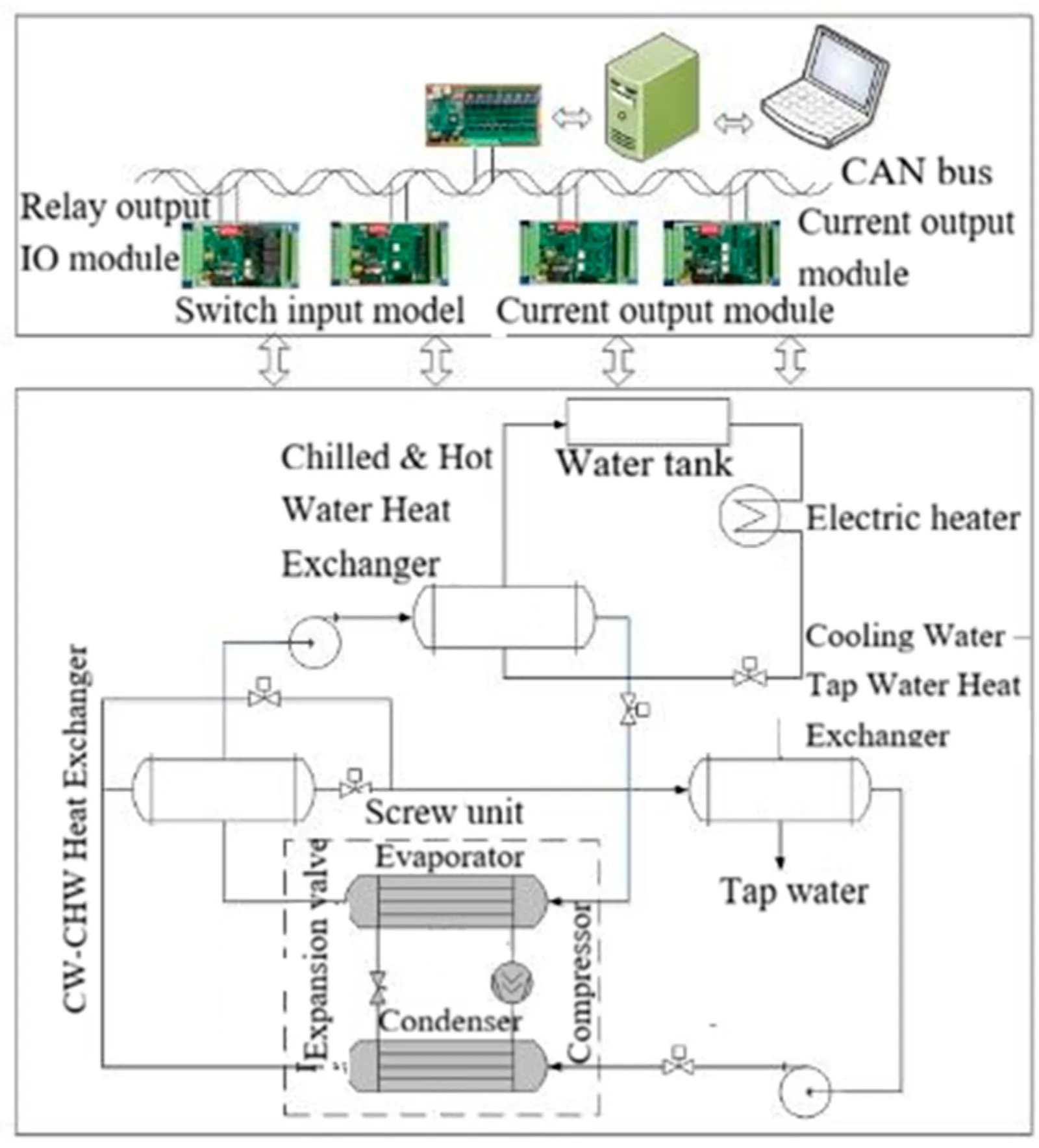
Schematic diagram of the cooling station experimental test bench.

The core equipment of the test bench is a screw chiller integrated with a compressor, condenser, electronic expansion valve, evaporator, supporting pipelines, and electronic control modules, all mounted on a shared base. The chiller adopts a return water constant-temperature control strategy. The data acquisition hardware includes multi-channel sensors for temperature, pressure, flow, current, and voltage as well as an acquisition card. The supporting upper computer software supports real-time data display, automatic storage, and batch post-processing. The layout of measuring points and fault simulation pipelines of the experimental system is illustrated in Figure 3.

In addition to the basic load regulation loop, additional fault simulation branches are installed on the platform, including suction–discharge bypass pipelines of the compressor, refrigerant charging regulating valves, and chilled/cooling water flow regulating valves. Sensor signals from all measuring points are transmitted to the PC via the acquisition card. The complete control logic of the chiller is described as follows: After power-on, the control system performs automatic self-inspection. If no fault is detected, the unit switches to automatic mode. The target water supply temperature is set on the touch screen before the chiller is started. The compressor adopts step-down start-up logic, and the unit adaptively adjusts its output power according to the real-time temperature of refrigerant water. All operating parameters and working states of the chiller can be checked in real time on the human–machine interaction interface. The rated operating standards of the unit are as follows: the outlet temperature of chilled water is kept constant at 7 °C, and the reference return temperature of cooling water is 20 °C.

#### 3.2.2. Operation Specifications for Five Types of Gradient Fault Simulation

**Figure 4:**
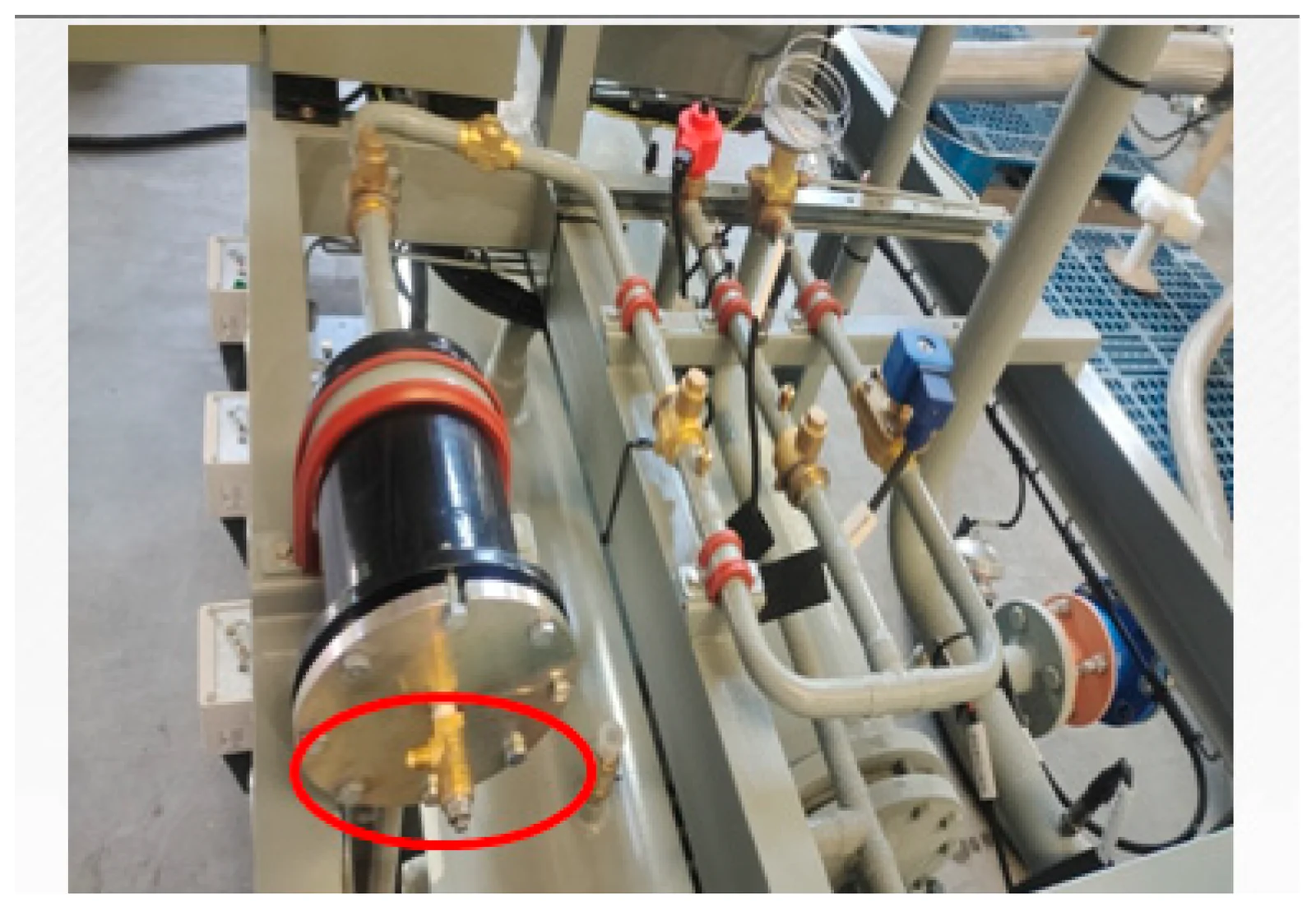
Charge refrigerant/nitrogen proportionally through the filling valve.

The platform can manually reproduce five categories of faults with three severity gradients. Together with the normal steady-state operating condition, a total of 16 refined operating states are covered. The regulation methods for each fault are specified as follows:

(1) Condenser cooling water flow degradation: Adjust the opening of the electric two-way valve for cooling water via the touch panel (fully open corresponds to 100% flow). The flow rates are set to 90%, 80%, and 70% of the rated value, respectively. After each adjustment, the unit operates steadily for a sufficient duration, and data are recorded only after all parameters stabilize.
(2) Chilled water flow degradation: Regulate the chilled water flow to 90%, 80%, and 70% of the rated working condition through the chilled water electric valve. Steady-state time-series samples are collected once the operating condition stabilizes.
(3) Excessive refrigerant charge: R134a refrigerant is injected in batches through the charging pipeline shown in Figure 4, with single injection amounts of 3 kg, 6 kg, and 9 kg, corresponding to overcharge gradients of 10%, 20%, and 30%. Data acquisition is conducted after stable operation following each injection.
(4) Insufficient refrigerant charge: The unit is fully evacuated first, then R134a is filled in batches with quantities of 18 kg, 22.5 kg, and 27 kg, equivalent to 60%, 80%, and 90% of the rated charge volume. Sensor data are recorded once the working condition becomes stable.
(5) Entrainment of non-condensable gas: Nitrogen is injected in batches via the charging branch displayed in Figure 4, with single injection weights of 3 kg and 6 kg, accounting for 10% and 20% of the standard total refrigerant mass. Samples are collected after steady operation.

After SG filtering, the curves of two early faults (excessive refrigerant and trace non-condensable gas) become nearly flat, which conforms to the physical laws of actual chiller operation. Slight refrigerant overcharge and a small amount of non-condensable gas only lead to extremely slow drift of condensing pressure without violent periodic parameter fluctuations. The SG filter adopted in this paper has a window length of 21, which smooths minor slow random noise and yields nearly horizontal curves. This characteristic precisely reflects the difficulty in identifying weak incipient faults of marine equipment and verifies the necessity of the hybrid attention mechanism proposed in this work for capturing subtle long-term drift features.

#### 3.2.3. List of Measuring Points for Target-Domain Data Acquisition

A total of 24 sensor acquisition channels are arranged on the test bench, covering temperature, pressure, flow, and electrical parameters. All measured variables are listed in Table 3.

#### 3.2.4. Unified Preprocessing Pipeline and Filtering Result Analysis

The target-domain data adopt exactly the same preprocessing pipeline as the source ASHRAE dataset, including outlier correction, SG smoothing filtering, and steady-state fragment screening, to guarantee unified processing rules for both datasets. The source domain is a public standard dataset with sufficient labels, a large total number of samples, and balanced operating conditions for all categories. In contrast, the target domain contains fewer labeled measured samples, and its time-series curves become generally smoother after identical smoothing treatment.

**Figure 5:**
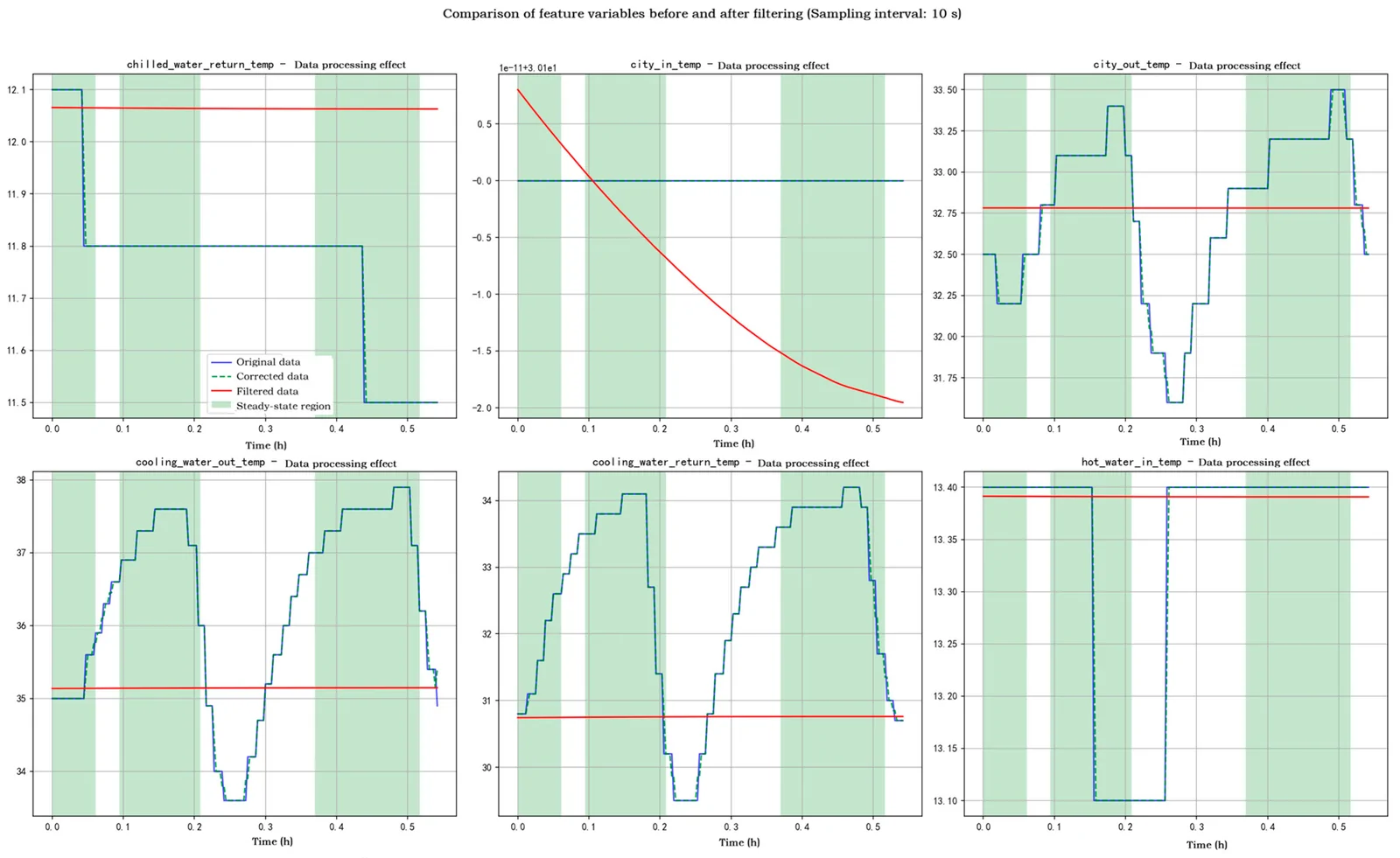
Filtered data curve under 10% reduction of chilled water flow.

Two typical gradient faults are selected to demonstrate the noise reduction performance of filtering: (1) chilled water flow degradation fault Taking the 10% flow reduction gradient as an example, the raw time series contains high-frequency jitters and random burrs. Noise is effectively suppressed after filtering and steady-state screening, and the comparison before and after processing is shown in Figure 5.

**Figure 6:**
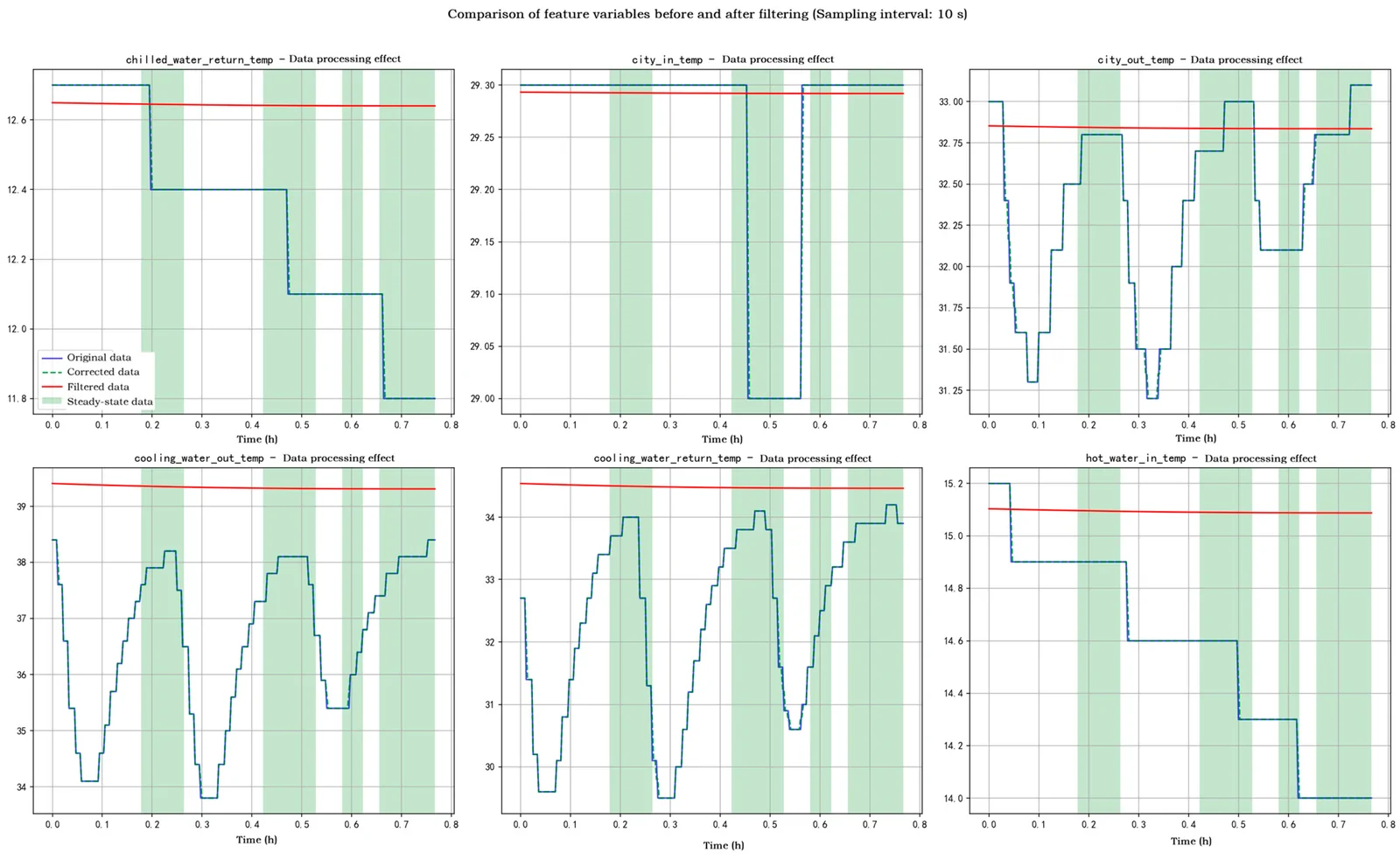
Filtered data curve under 10% insufficient refrigerant charge.

(2) Insufficient refrigerant charge fault Taking the operating condition with a 10% refrigerant shortage as an example, the filtering eliminates random sensor disturbances while retaining the inherent slow drift features of the fault. The processing results are presented in Figure 6.

In the weighted cross-entropy loss function of the classifier proposed in this paper, the loss weight assigned to the refrigerant overcharge (RO) category is smaller than that for refrigerant lack (RL), which is determined by the measured data characteristics and sample distribution of the two faults. Insufficient refrigerant leads to persistent abnormal heat exchange in the evaporator, accompanied by obvious periodic large fluctuations in temperature and pressure time series, making its fault features prominent and easy for the model to identify. By contrast, the unit operates within a narrow steady-state range for a long time under slight refrigerant overcharge; sensor parameters only exhibit extremely slow and tiny drifts, resulting in a higher proportion of hard-to-distinguish samples with low fault identifiability. To prevent the model from biasing toward simple and easily separable faults, a smaller loss weight is configured for the harder-to-identify overcharge condition to balance the gradient contribution during training.

## 4. Model Training and Detailed Settings

This section analyzes the data characteristics of chiller datasets in both source and target domains and elaborates in detail on the drawbacks of the original DDPN model, the five-stage iterative training scheme proposed in this paper, the design of multi-loss functions, and the overall optimization logic. To address challenges including heterogeneous equipment between source and target domains, numerous fault types, finely divided fault severity levels, scarce labeled samples in the target domain, and imbalanced sample distribution, a staged training strategy and a joint multi-loss optimization mechanism are constructed. This ensures stable model training and reliable diagnostic performance under cross-equipment and few-shot conditions.

### 4.1. Limitation Analysis of the Original DDPN Model

**Figure 7:**
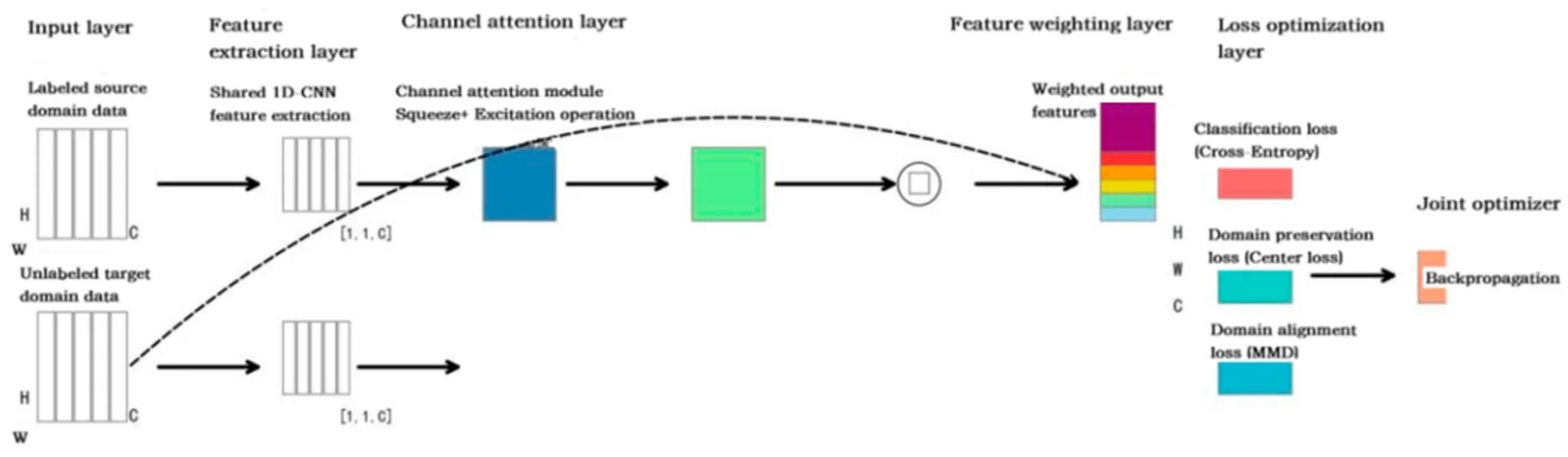
Working flowchart of the original DDPN.

The original DDPN is a classic cross-domain transfer learning model. Its core idea lies in jointly executing feature extraction, domain preservation, and domain alignment tasks. It leverages methods such as maximum mean discrepancy (MMD) to narrow the distribution discrepancy of data across different domains, enabling preliminary adaptation to industrial fault diagnosis scenarios with variable operating conditions, few labeled samples, and unlabeled data. Its basic workflow is illustrated in Figure 7.

Considering the practical application scenarios of shipboard chillers in this paper, such as severe cross-equipment domain shift, finely divided fault severity levels, noisy measured data, and scarce labeled target samples, the original DDPN model has prominent shortcomings: 1. Insufficient domain alignment performance: The original DDPN only adopts a single non-adversarial maximum mean discrepancy (MMD) loss to realize domain alignment. When confronted with substantial equipment heterogeneity between the ASHRAE centrifugal chiller and the laboratory screw chiller as well as drastic feature distribution shifts, it achieves limited feature alignment effects. 2. High risk of feature overfitting: The domain preservation module excessively enhances source-domain feature representation, which reduces the generalization ability when the model is transferred to the target chiller and readily causes overfitting. 3. Unstable training process: Conflicts exist among the three joint loss optimization objectives. Coupled multi-task training tends to trigger gradient oscillation, resulting in poor training performance, particularly on few-shot datasets. 4. Weak anti-noise capacity: No targeted optimization is designed for noisy sensor data collected from actual operating sites, so the model cannot adapt well to engineering-measured data. 5. Limited scenario compatibility: It merely supports basic closed-set recognition and fails to satisfy the diagnostic demands of multi-category faults with graded severities studied in this work.

To remedy the above deficiencies, this paper comprehensively optimizes and upgrades the original DDPN model. By integrating a hybrid attention mechanism, residual structure, adversarial domain adaptation, and semi-supervised learning techniques, a five-stage iterative training strategy and a staged activation scheme for multiple loss functions are devised. A dual-hybrid attention feature discriminant domain-adversarial network (DAFDAN) is established to achieve precise fault diagnosis for chillers with multiple fault severities across different equipment platforms.

### 4.2. Five-Stage Segmented Training Strategy

In accordance with dataset characteristics, the source domain adopts the ASHRAE RP-1043 centrifugal chiller dataset, covering five typical faults: reduced evaporator water flow, reduced condenser water flow, insufficient refrigerant, excessive refrigerant, and infiltration of non-condensable gas. Each fault contains multiple severity levels with sufficient fully labeled samples. The target domain consists of measured data from a laboratory screw chiller, which involves the same five fault types. Each fault is set with three severity gradients of 10%, 20%, and 30%, forming a total of 16 operating states. The target domain only has a small number of labeled samples, representing a typical few-shot scenario.

To fully exploit the abundant prior knowledge of the source domain and progressively adapt to heterogeneous target domain data and subdivided faults, a five-stage progressive iterative training scheme is proposed in this paper. During training, network modules are dynamically frozen or unfrozen, and corresponding loss functions are activated stage by stage to realize knowledge transfer and model adaptation in a step-by-step manner from shallow to deep levels.

Stage 1: Source domain pre-training. Relying on massive, labeled source-domain data, this stage implements basic training for the source-domain encoder and multi-granularity classifier to learn universal fault features and classification rules of chillers. Meanwhile, MMD loss is adopted to preliminarily constrain the feature distributions of source and target domains.

Stage 2: Warm-up of the target domain extender. Under unsupervised mode, this stage initializes the parameters of the target domain extender, mapping heterogeneous features into a unified dimensional space to address feature heterogeneity caused by different sensor configurations of the two types of chillers.

Stage 3: Adversarial domain adaptation training. All network modules are unfrozen, and three loss functions, including CE, BCE, and MMD, are activated simultaneously. This stage performs adversarial learning combining the gradient reversal layer (GRL) and domain discriminator. Cooperating with MMD loss, it narrows domain shift through dual constraints of implicit adversarial alignment and explicit distance metric so as to reduce feature distribution deviation induced by equipment differences.

Stage 4: Semi-supervised consistency training. The entire network is set trainable, and the KL divergence loss is additionally introduced for joint optimization. This stage exploits the latent value of abundant unlabeled target-domain data. Consistency regularization is utilized to boost model robustness and further alleviate the shortage of labeled samples in the target domain.

Stage 5: Few-shot fine-tuning. All five loss functions are weighted for joint optimization. The accuracy on the target validation set is monitored throughout the training process, and the training will terminate in advance once the early-stopping condition is satisfied. A small number of labeled target-domain samples are used for global fine-tuning to adapt the model to the 16 subdivided fault states of the target chiller. An early stopping strategy is applied simultaneously to suppress overfitting.

### 4.3. Multi-Loss Joint Optimization Function

This paper differentially activates five loss functions, including weighted cross-entropy ($L_{CE}$), maximum mean discrepancy (MMD) loss ($L_{MMD}$), mean square error (MSE) reconstruction loss ($L_{MSE}$), binary cross-entropy (BCE) adversarial loss ($L_{BCE}$), and Kullback–Leibler (KL) divergence loss ($L_{KL}$), according to the distinct tasks of the five-stage training so as to establish a multi-constraint joint optimization framework. The mathematical formula, design motivation, and applicable training stage of each loss function are elaborated as follows.

(1) Weighted Cross-Entropy Loss $L_{CE}$

As the core classification loss of the model, weighted cross-entropy loss is adopted throughout the 1st, 3rd, 4th, and 5th training stages. Imbalanced fault sample quantities exist in both source and target domains. Therefore, category weights are assigned to each fault class to boost the identification performance of low-proportion and low-severity faults, realizing precise classification of 5 major fault categories and 16 subcategories. Its expression is given by the following:

$$L_{CE}=-\sum_{i=1}^{N}q_{i}\log(p_{i})$$

where p denotes the predicted probability distribution output by the model, q represents the true label distribution of samples, and N is the total number of samples.

(2) Maximum Mean Discrepancy (MMD) $L_{MMD}$

LossMMD explicitly measures the Euclidean distance between the feature distributions of the source and target domains. It is mainly adopted in Stage 1, Stage 3, Stage 4, and Stage 5 and cooperates with adversarial loss to realize two-layer domain alignment. Its expression is shown as follows:

$$L_{MMD}={\left\|E[z_{src}]-E[z_{tgt}]\right\|}_{2}^{2}$$

where zsrc and ztgt stand for the shared features of the source domain and target domain, respectively, and E denotes the expectation of features.

(3) Mean Squared Error (MSE) Reconstruction Loss $L_{MSE}$

The MSE reconstruction loss is only activated in Stage 2 for the warm-up of the target domain extender. As an unsupervised loss, it constrains the encoding and reconstruction of target domain features to guarantee intact information after heterogeneous feature mapping. Its formula is given below:

$$L_{MSE}=\frac{1}{N}\sum_{i=1}^{N}(x_{i}-{\hat{x}}_{i}{)}^{2}$$

where x is the raw feature of the target domain, and x^ denotes the reconstructed feature output from the network.

(4) Binary Cross-Entropy (BCE) $L_{BCE}$

Adversarial Loss: The BCE loss serves as the optimization objective of the domain discriminator, applied in Stages 3 and 4. Implicit domain alignment is achieved through adversarial game training to endow features with domain-irrelevant attributes. Its formula is shown as follows:

$$L_{BCE}=-\frac{1}{N}\sum_{i=1}^{N}\left[y_{i}\log(p_{i})+(1-y_{i})\log(1-p_{i})\right]$$

where p is the prediction output of the domain discriminator, and y represents the domain label.

(5) KL Divergence Consistency $L_{KL}$

LossKL divergence loss is only activated for semi-supervised training in Stage 4. It constrains the output distribution consistency between original samples and noisy samples and leverages unlabeled target-domain data to improve the generalization capacity of the model. Its mathematical expression is as follows:

$$L_{KL}=\sum_{i=1}^{N}P_{i}\log\left(\frac{P_{i}}{Q_{i}}\right)$$

where P and Q refer to the predicted distributions of original samples and noise-added samples, respectively.

The five types of losses differ drastically in physical meaning and value ranges: the classification loss LCE and adversarial loss LBCE are mainly distributed within [0, 1], while the MMD distance loss LMMD and MSE reconstruction loss LMSE can reach values of dozens. The overall magnitude of KL divergence loss LKL remains relatively small. Direct equal-weight summation will make losses with larger magnitudes dominate gradient updating entirely, leading to imbalance among classification, domain alignment, and semi-supervised learning tasks.

This paper adopts a stage-wise differentiated weighted summation strategy. A fixed set of balance weights $\lambda_{CE}$, $\lambda_{MMD}$, $\lambda_{MSE}$, $\lambda_{BCE}$, and $\lambda_{KL}$ is assigned for each training stage to unify the contribution magnitude of each loss via scaling coefficients. The total stage-wise loss is formulated as follows:

$${L_{total}=\lambda_{CE}L}_{CE}+{\lambda_{BCE}L}_{BCE}+{\lambda_{MMD}L}_{MMD}+\lambda_{MSE}L_{MSE}+\lambda_{KL}L_{KL}$$

where λ denotes the balance weight of the corresponding loss. Fixed weights are assigned to losses activated in the current stage, while weights of inactive losses are set to 0 to automatically eliminate such constraint terms.

Activation rules and weight configurations of losses across stages:

Stage 1 (Source-domain pre-training): Only weighted cross-entropy loss is activated.

$${L_{total}^{1}=1.0L}_{CE},\lambda_{MMD}=\lambda_{MSE}=\lambda_{BCE}=\lambda_{KL}=0$$

Stage 2 (Target extender warm-up): Only MSE reconstruction loss is activated.

$${L_{total}^{2}=0.8L}_{MSE},\;\text{all}\;\text{other}\;\text{weights}\;\text{equal}\;0.$$

Stage 3 (Adversarial adaptive training): Classification, MMD, and adversarial losses are activated.

$${L_{total}^{3}=1.0L}_{CE}+{0.6L}_{BCE}+{0.3L}_{MMD},\lambda_{MSE}=\lambda_{KL}=0$$

Stage 4 (Semi-supervised consistency training): KL divergence constraint is additionally introduced.

$${L_{total}^{4}=1.0L}_{CE}+{0.6L}_{BCE}+{0.3L}_{MMD}+0.2L_{KL},\lambda_{MSE}=0$$

Stage 5 (Global fine-tuning with joint optimization of all losses): All five losses participate in weighted optimization simultaneously.

$${L_{total}^{5}=1.0L}_{CE}+{0.6L}_{BCE}+0.1{L_{MSE}+0.3L}_{MMD}+0.2L_{KL}$$

Explanation of the weighting mechanism: since MMD and MSE losses naturally yield large numerical values, relatively small weights (0.1/0.3) are configured to restrain their dominance over gradients. The core classification task is assigned the maximum weight of 1.0 to prioritize fault identification as the primary optimization objective. Adversarial, semi-supervised, and reconstruction losses adopt moderate weights and serve as auxiliary constraints to participate in training in a balanced manner. Irrelevant losses are dynamically masked stage by stage throughout the training process; each step only optimizes tasks corresponding to the current stage, thus avoiding gradient conflicts among multiple tasks.

### 4.4. General Hyperparameter Settings

Global hyperparameters are uniformly set based on dataset characteristics and the rules of five-stage training. All comparison models and ablation models adopt identical configurations to ensure experimental fairness:

Base optimizer: The Adam optimizer is selected to adapt to the five-stage layered training framework proposed in this paper. During joint optimization with multiple loss functions, the gradient directions fluctuate frequently. The adaptive learning rate of Adam can effectively suppress gradient oscillation. For cross-domain tasks with limited labeled samples, Adam achieves far more stable convergence than conventional fixed-learning-rate optimizers such as SGD, with an initial global learning rate of 1 × 10^−4^;

Batch Size: 32;

Maximum training epochs: 100 Epochs;

Early stopping criterion: Training terminates if the loss on the validation set fails to decrease for 5 consecutive epochs to avoid overfitting;

Gradient reversal coefficient α: 0.1;

Unified feature dimension: 30 dimensions;

Batch normalization, hybrid attention mechanism, and residual connections for network modules are enabled by default.

The fixed configuration of loss balance weights for each stage is shown in Table 4.

Dataset partitioning rules: All samples from the source ASHRAE dataset are split into training, validation, and test sets at a ratio of 7:1:2. All time-series samples of the target domain are divided into a 70% training set, a 15% validation set, and a 15% test set. Only 12% of the training samples in the target domain are labeled fault data, while the remaining unlabeled samples are adopted for semi-supervised KL training. Early stopping strategy: The total loss on the target validation set is calculated after each epoch. If the validation loss does not decrease for five consecutive epochs, the model is considered converged, and the iteration will be terminated in advance to avoid overfitting on small-scale samples.

## 5. Fault Diagnosis Research

### 5.1. Evaluation Metrics

For model performance evaluation, accuracy, F1-score, and confusion matrix are adopted as fundamental metrics in this paper. Meanwhile, to guarantee statistical validity, all experiments are independently repeated five times. The mean and standard deviation of each group of metrics are recorded for inter-group significance analysis. The mean value represents the average performance across repeated runs and reflects the stable recognition capacity of the model, while the standard deviation quantifies the fluctuation of experimental results—a smaller standard deviation indicates stronger generalization robustness of the model. Combined with the two quantitative metrics and statistical test outcomes, the cross-equipment fault diagnosis capability of the model can be comprehensively evaluated from multiple dimensions, including overall accuracy, class balance, fine-grained identification, and statistical reliability.

### 5.2. Analysis of Experimental Results

The enhanced DDPN model proposed in this paper adapts from general scenarios to the target domain in a progressive manner via a staged learning strategy, which consists of source domain pre-training, target domain extender warm-up, adversarial alignment, semi-supervised training, and few-shot fine-tuning. This approach avoids the instability inherent in end-to-end joint training. Meanwhile, three complementary domain adaptation mechanisms—maximum mean discrepancy (MMD) mean alignment, gradient reversal adversarial training, and semi-supervised consistency constraints—are integrated to narrow the cross-device distribution discrepancies from the perspectives of statistics, adversarial training, and prediction accuracy. In the feature extraction module, a source domain compressor with unified output dimensions and a target domain extender embedded with attention mechanisms and residual connections are adopted, together with a shared classifier, to realize knowledge transfer. Furthermore, auxiliary classification loss, class-weighted cross-entropy, and validation-set-based early stopping are applied to further improve feature stability and mitigate the challenges posed by few-shot learning and class imbalance.

#### 5.2.1. Model Validation and Training Loss

**Figure 8:**
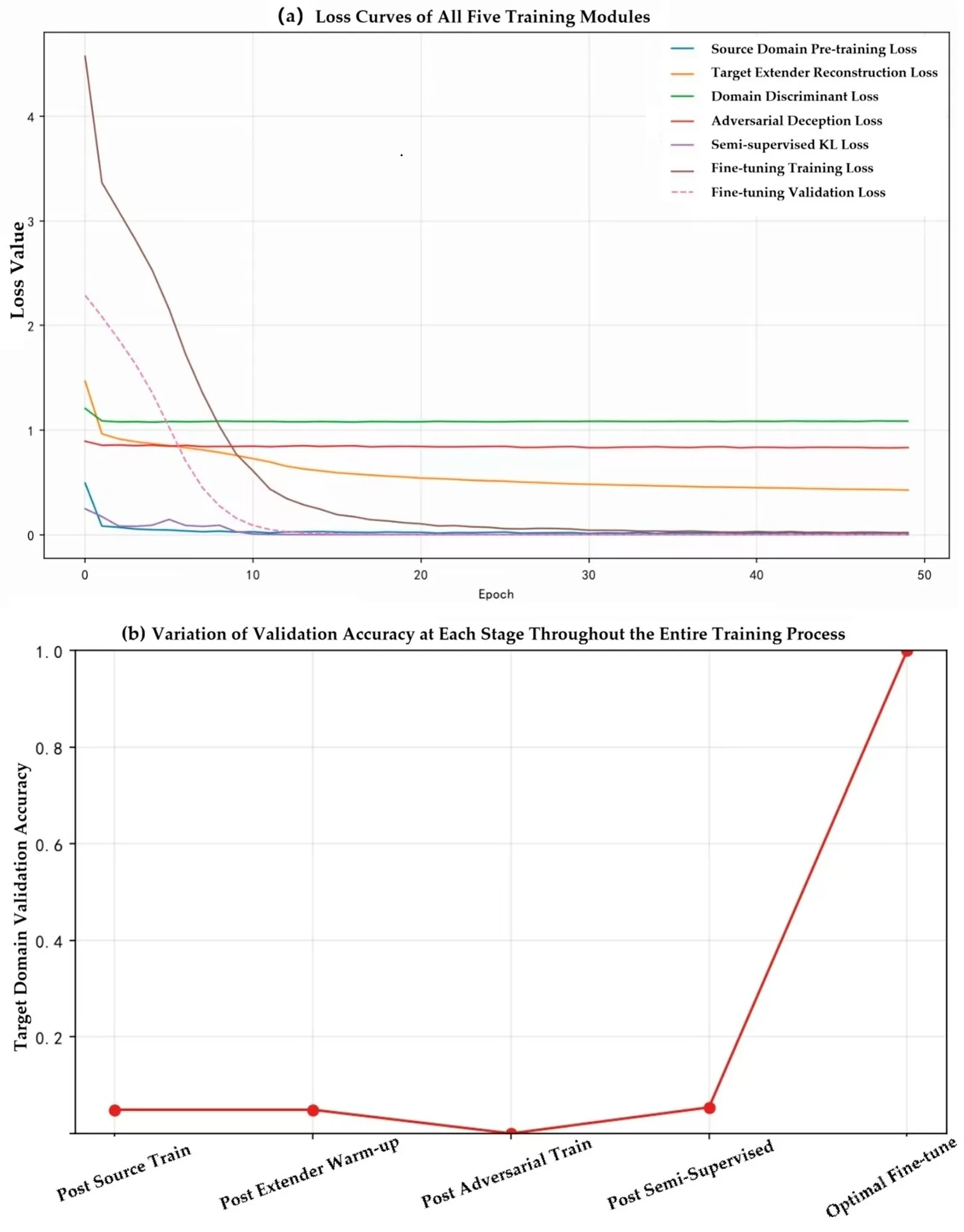
Multi-stage Loss Curves.

To intuitively demonstrate the complete five-stage training dynamics of DAFDAN, this paper integrates the scattered multi-stage loss curves into Figure 8, which consists of two subgraphs. Figure 8a plots the variation curves of all losses from the five major training modules against training epochs, including six curves: source pre-training classification loss, target extender MSE reconstruction loss, domain discriminator adversarial loss, semi-supervised KL consistency loss, total loss during global fine-tuning, and validation loss in the fine-tuning phase. Figure 8b presents the line chart of target-domain validation accuracy across separate training stages. Its horizontal axis corresponds to the full five-stage training pipeline of the model: source pre-training → extender warm-up → adversarial training → semi-supervised optimization → global fine-tuning.

Intuitive training laws can be summarized from the loss convergence characteristics in Figure 8a: at the initial training epoch (Epoch = 0), all losses start at high values. As iterations proceed, all loss terms decline rapidly without violent curve fluctuations. This verifies that the proposed multi-loss joint training scheme combining stage-wise loss activation and differentiated weighting effectively alleviates gradient conflicts among multiple optimization objectives.

The source pre-training loss (blue curve) converges rapidly to near zero within the first 10 epochs, demonstrating that abundant labeled source samples enable fast fitting of basic fault features. The target extender reconstruction loss (orange curve) drops sharply in the early stage and decreases slightly and slowly afterwards, completing the warm-up mapping for feature spaces of heterogeneous screw chillers. The adversarial discrimination loss (green curve) remains within a small and stable value range throughout training, continuously constraining distribution alignment between source and target domains without converging fully to zero. The semi-supervised KL loss (purple curve) maintains an extremely low magnitude all the time, smoothing the decision boundaries of fine-grained faults by utilizing unlabeled samples. The total loss during global fine-tuning (brown curve) exhibits the largest decreasing amplitude and approaches zero by the end of the 50th epoch. It converges synchronously with the fine-tuning validation loss (pink dashed line), and no obvious overfitting is observed.

The target-domain validation accuracy curve in Figure 8b visually reveals the layered optimization effect of stage-wise training: when only source pre-training and target extender warm-up are finished, the target validation accuracy remains at an extremely low level. At this point, the model only learns feature patterns of source centrifugal chillers and possesses no cross-equipment generalization ability at all. Upon entering the adversarial training stage, the accuracy slightly drops, which is a normal oscillation caused by the domain confusion constraint introduced via the gradient reversal layer. The accuracy recovers gradually in the semi-supervised optimization stage. After the global fine-tuning in Stage 5, the target validation accuracy surges to nearly 100%. This outcome proves that the complete collaborative multi-loss constraints and five-stage layered training pipeline are essential prerequisites for high-precision cross-domain fault diagnosis.

#### 5.2.2. Ablation Experiments

To fully demonstrate the individual necessity of the hybrid attention module, multi-loss joint optimization, and five-stage progressive training strategy proposed in this paper, three groups of independent controlled-variable ablation experiments are designed in this section. An ablation experiment refers to a set of controlled comparative experiments whose core idea is to remove only one core module of the model at a time while keeping the rest of the architecture unchanged. The independent contribution of each module to the overall model is quantified through performance variations.

All experimental groups adopt identical basic network architectures, dataset partitioning schemes, and iterative hyperparameters, with only a single variable modified in each group. Each experiment is independently repeated five times, and two sets of evaluation metrics for the source domain and target domain are output simultaneously. The accuracy, F1-score, and standard deviation on the unseen target test set are taken as the core evaluation criteria.

##### Ablation of Core Network Modules

In this group of experiments, all five loss functions and the complete five-stage training pipeline are retained unchanged. Only three major network modules, namely the hybrid attention mechanism, adversarial training branch, and semi-supervised learning component, are removed one by one. The quantitative results are presented in Table 5.

The data in Table 5 clearly illustrates the misleading performance metrics caused by removing the adversarial module. After eliminating adversarial training, the source-domain accuracy and F1-score reach the highest values of 99.39% in the table. However, this high score merely indicates that the model over-memorizes unique features of source-domain centrifugal chillers. After removing the hybrid attention module, noise cannot be filtered out, and the subtle fault features corresponding to 10% and 20% degradation levels are obscured, resulting in a sharp drop in the target-domain accuracy to 77.93%. Without the dual domain alignment constraints of MMD and BCE losses, the model fails to mitigate multi-layer distribution shifts between the two types of equipment, resulting in complete loss of cross-equipment generalization and a sharp drop of target-domain diagnostic accuracy to 68.74%. This directly verifies that the adversarial branch is an indispensable network component for eliminating cross-domain discrepancies.

Results from the other two ablation groups further reveal that removing the hybrid attention mechanism simultaneously degrades recognition accuracy on both source and target domains, as the model’s ability to resist noise and extract fine-grained features deteriorates drastically. When the semi-supervised module is discarded, the target-domain F1-score decreases substantially owing to the loss of feature regularization provided by unlabeled samples. Collectively, these findings demonstrate that the three core network modules are all essential; any omission will lead to a notable decline in cross-domain fault diagnosis performance.

##### Ablation of Different Loss Combinations

In this set of experiments, the complete network architecture and five-stage training strategy are fixed. Only one loss function among MMD, BCE, MSE, and KL is excluded each time to quantitatively verify the independent structural value of each loss term. The results are listed in Table 6.

The quantitative results in Table 6 allow separate analysis of the irreplaceable function of each loss term. Eliminating any one of the four auxiliary losses (MMD, BCE, MSE, KL) leads to a sharp plummet in target-domain accuracy. Only adopting MMD merely matches the first-order mean value and fails to calibrate covariance and high-order entropy. The BCE-based adversarial loss ignores the global statistical distance. The absence of MSE leads to distorted mapping of heterogeneous features, and removing KL loss makes full use of unlabeled samples impossible. A single loss function can only constrain distribution discrepancies at a single level and cannot simultaneously handle four types of coupled shifts. Therefore, the synergistic effect of multiple losses is indispensable. If merely the basic classification loss is adopted without any domain adaptation constraints, the accuracy is only 58.30%. Specifically, MMD realizes global feature mean matching, BCE achieves implicit domain adversarial confusion, MSE completes unsupervised feature mapping for heterogeneous screw chillers, and KL refines fault classification boundaries using unlabeled samples. The four loss terms complement each other with distinct responsibilities; the absence of any single term prevents thorough elimination of multi-layer domain shifts. While the loss convergence curves in Figure 8 merely reflect the iterative training process, the quantitative metrics under various loss combinations in this table constitute the core quantitative evidence verifying the independent structural significance of each loss function.

##### Ablation of Five-Stage Progressive Training Strategy

In this group of experiments, the complete network architecture and all five loss functions are kept fixed. Only two training paradigms are compared: the proposed five-stage progressive training and the conventional single-stage end-to-end synchronous training to validate the necessity of the layered training scheme. The quantitative results are summarized in Table 7.

With identical network and loss configurations, simplifying the pipeline into single-stage synchronous training causes the target-domain accuracy to plummet from 99.98% to 68.74%. In single-stage end-to-end synchronous training, the gradients of the five loss functions counteract each other. The feature extractor cannot balance multiple objectives, including classification, domain alignment, and feature reconstruction, simultaneously. As a result, the model rapidly overfits the source-domain data and loses its generalization capability across different units. In contrast, the proposed five-stage layered strategy decomposes optimization tasks step by step. It freezes and unfreezes network layers in phases and activates corresponding loss functions sequentially, progressively narrowing the feature information entropy gap between the source and target domains. Only full implementation of the five-stage pipeline can achieve nearly perfect recognition accuracy.

A unified core conclusion can be drawn from the three groups of ablation experiments: severe compound domain shifts exist between the source centrifugal chillers and target marine screw chillers, stemming from disparities in equipment structures, sensor dimensions, operating noise, and labeled sample distributions. The experimental task is highly complex rather than a simple dataset scenario. Only the complete DAFDAN framework integrated with the hybrid attention module, the full set of five complementary loss functions, and the five-stage layered training strategy can deliver nearly 100% diagnostic accuracy on the unseen target test set. Removing any core network module, eliminating any single loss term, or simplifying the layered training pipeline will result in a precipitous drop in target-domain recognition performance. While training loss convergence curves only illustrate the superficial iterative process of model training, the three multi-dimensional quantitative ablation tables in this section serve as pivotal quantitative evidence validating the irreplaceability of the multi-loss coordination mechanism and five-stage training strategy.

#### 5.2.3. Comparative Experiments

To further verify the superiority of the proposed method, we compare it with several classic transfer learning and non-transfer learning approaches, which are introduced as follows:

SVM: A traditional support vector machine model trained on source domain data.

DNN: A deep neural network model trained on source domain data.

MMD: A deep transfer learning method that adopts only the MMD loss for domain alignment.

DANN: The classic domain adversarial neural network.

CORAL: A domain adaptation method based on covariance matrix alignment.

DAFDAN (proposed method): The improved model presented in this paper.

For all multi-model comparison experiments in this section involving SVM, DNN, MMD, CORAL, DANN, and DAFDAN, the average accuracy is uniformly calculated based on an independent target-domain test set, and the reported metrics are the mean values obtained from five experiments with random dataset splits. The source domain is only utilized for model pre-training and excluded from cross-domain accuracy statistics. Training sets and unlabeled samples are merely adopted for parameter optimization and not included in the final evaluation.

**Figure 9:**
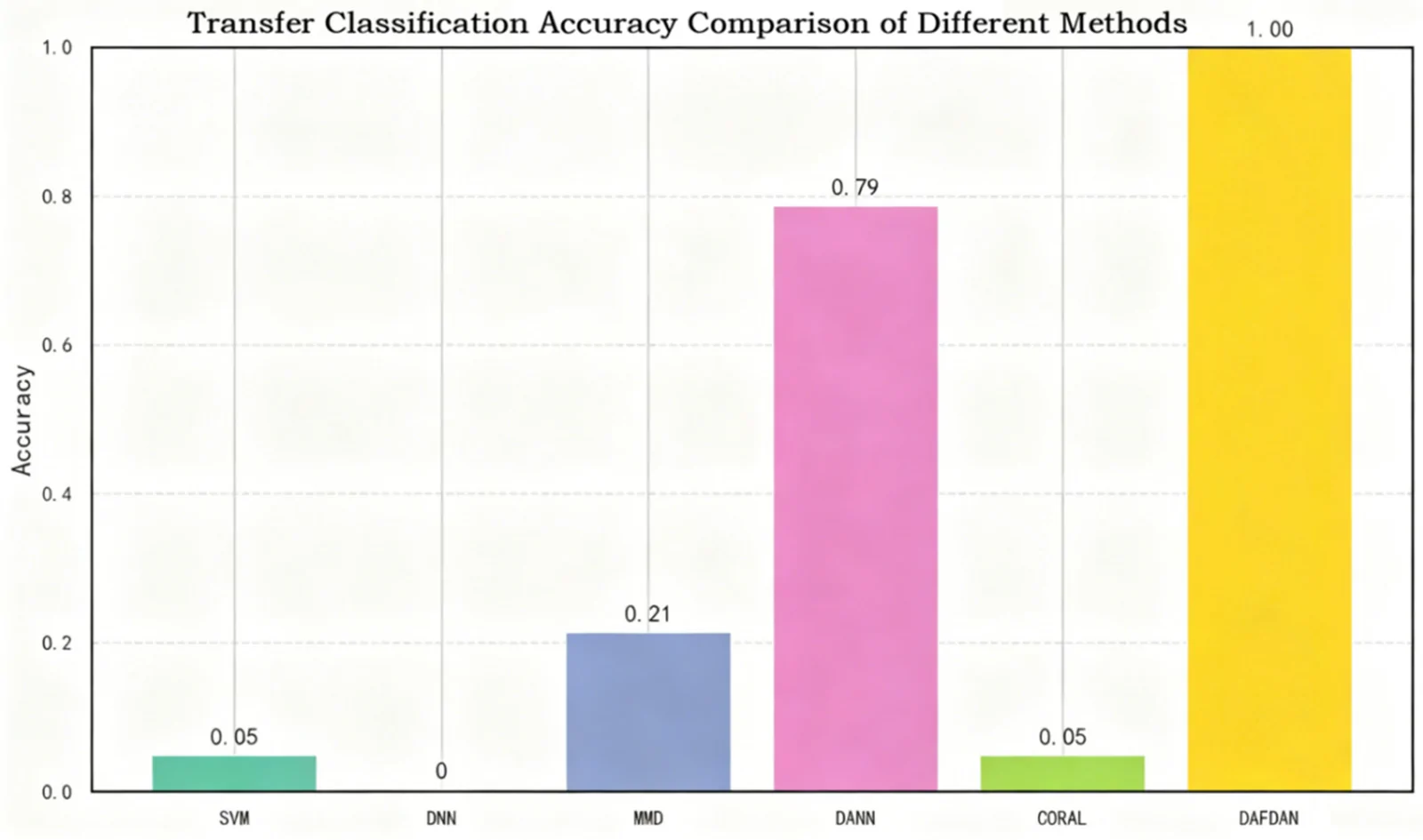
Comparison of Transfer Classification Accuracy of Different Methods.

The classification accuracy of the six algorithms for cross-device fault transfer diagnosis of chilled water units is presented in Figure 9. The vertical axis represents the fault recognition accuracy ranging from 0 to 1. All models are trained solely on source domain samples and tested directly on unexposed experimental data from the target domain, so as to evaluate their cross-domain generalization ability.

SVM and DNN are traditional models without domain adaptation modules. Trained only on source domain data and lacking cross-domain distribution correction, they deliver poor performance on the target domain. The accuracy of DNN is close to zero, which means it fails to realize cross-device fault diagnosis. Deep neural networks tend to overfit operating-condition-specific features of the source domain and cannot extract universal fault features of chilled water units. Severe domain shift occurs when processing target domain data, leading to complete model failure. The accuracy of SVM is merely 0.05, which is equivalent to random guessing. Traditional machine learning methods rely on manually designed features and have no distribution adaptation capability. The differences in operating conditions and sensor data between the source and target domains will break the classification hyperplane and result in a total loss of generalization ability. The two baseline models fully prove that models without domain adaptation strategies cannot achieve effective transfer diagnosis for cross-device fault diagnosis of ship chilled water units.

MMD and CORAL are classic transfer learning algorithms based on statistical distribution alignment, which reduce distribution discrepancies by matching inter-domain statistics but achieve limited performance improvement. The accuracy of MMD is 0.21, a slight increase compared with the non-transfer baselines. Maximum mean discrepancy only constrains the overall distance between feature distributions. It cannot distinguish discriminative fault features from device-related interference features and can only roughly align global distributions, making it difficult to extract fine-grained fault representations. Hence, its diagnosis accuracy has an obvious bottleneck. CORAL achieves an accuracy of only 0.05, on a par with SVM. As it merely matches second-order statistics, CORAL cannot eliminate high-order feature differences between domains when dealing with multi-dimensional and strongly coupled sensor time-series data of chilled water units. Without a feature screening mechanism, operating-condition noise severely interferes with covariance alignment, so the method is inapplicable to fault transfer tasks for complex refrigeration systems. The results indicate that shallow domain adaptation strategies relying purely on global statistic alignment are incapable of addressing the complex domain shift caused by multi-level faults and coupled multi-sensor data and suffer from insufficient capability to extract discriminative fault features.

As a classic adversarial domain adaptation network, DANN adopts adversarial game to realize implicit domain distribution correction and achieves an accuracy of 0.79, a substantial improvement over statistical alignment methods. Through the gradient reversal layer, adversarial training mixes features from the source and target domains, weakens device-specific operating features, and preserves shared fault features, thereby greatly alleviating cross-device domain shift. Nevertheless, DANN has two major drawbacks. First, it lacks a feature focusing mechanism and treats irrelevant noise and key fault sensor signals equally. Mixed multi-dimensional data related to chilled water, cooling water, and refrigerant interferes with adversarial learning. Second, it adopts only a single adversarial loss for distribution constraints and is not equipped with semi-supervised consistency regularization. It cannot utilize unlabeled target domain samples to optimize classification boundaries, leaving a diagnosis error of about 21%.

The proposed DAFDAN achieves a cross-device fault transfer classification accuracy of nearly 100%, outperforming SVM, MMD, DANN, CORAL, and other comparative algorithms comprehensively. Its superior performance benefits from the synergistic effect of three innovative mechanisms: hybrid attention-based feature screening, multi-loss hierarchical domain adaptation, and semi-supervised KL regularization.

#### 5.2.4. Confusion Matrix and Category Performance Analysis

Confusion matrices are adopted in this section to visualize the fine-grained fault identification performance of the model. Although the overall diagnostic accuracy of the model on the target domain reaches 99.98%, relying solely on a single global average accuracy metric has obvious limitations and cannot fully verify the model’s hierarchical diagnosis capability. Global accuracy only calculates the average recognition correctness of all samples, which may lead to a high overall score accompanied by frequent misclassification between similar faults and faults with varying severity levels. Such a shortcoming fails to meet the engineering requirements of refined operation, maintenance, and hierarchical early warning for marine chillers.

**Figure 10:**
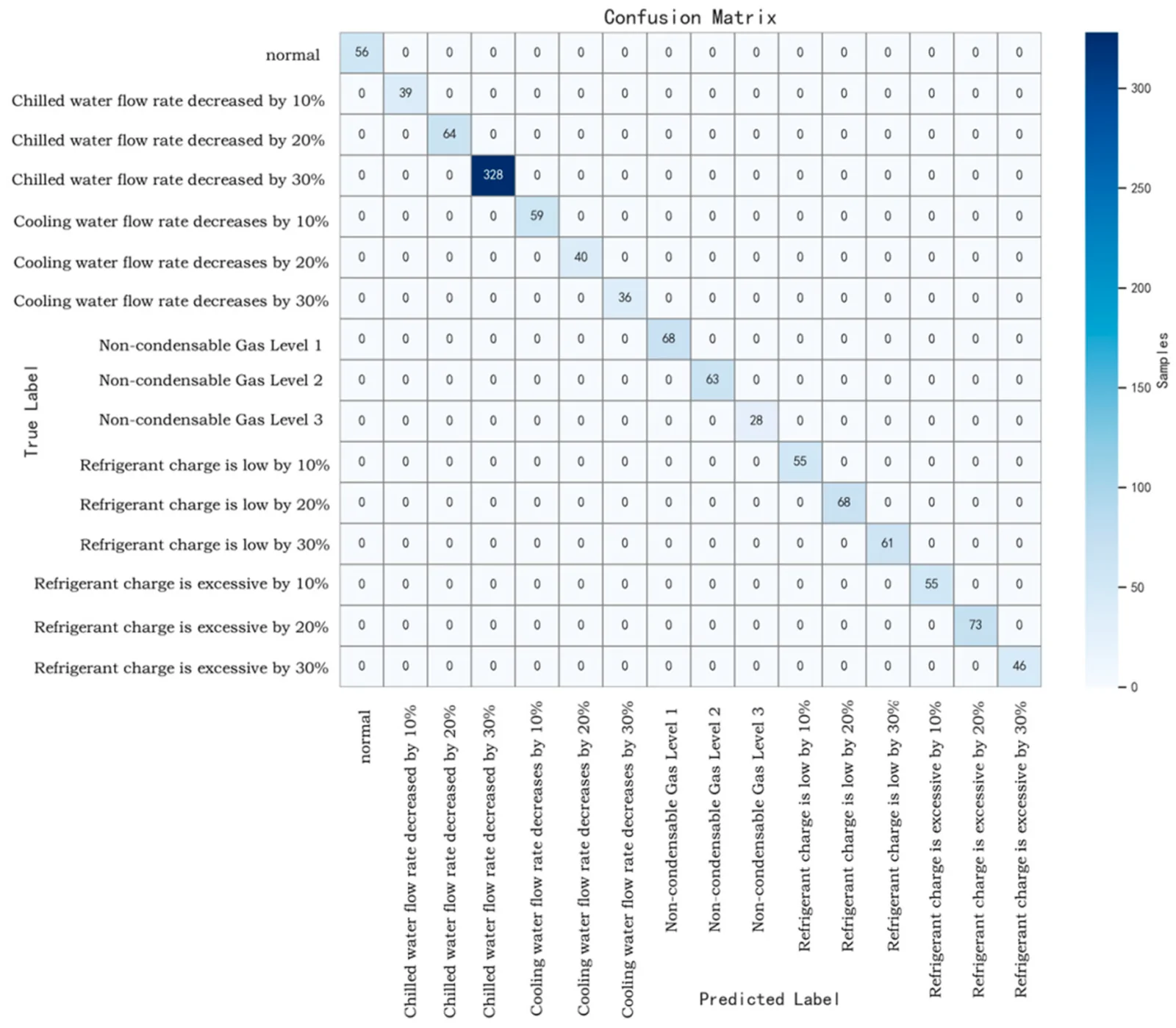
Confusion Matrix of Fault Classification for DAFDAN on Target Test Set.

Figure 10 covers all 16 fine-grained operating conditions and intuitively demonstrates the independent classification boundary of each fault, supplementing the fine-grained information missing from the global accuracy from a visualization perspective. It can be observed from the matrix that all samples fall on the diagonal without any off-diagonal misclassified samples. Specifically, no false alarms occur for normal steady-state samples; there is no internal confusion among the three gradient levels of chilled/cooling water flow degradation; two highly similar faults of refrigerant undercharging and overcharging are completely distinguished; and subtle latent faults of non-condensable gas at levels 1 to 3 can be accurately identified, with no confusion between minor faults and normal operating conditions.

To quantitatively verify the zero-misclassification effect shown in the confusion matrix, the individual precision and recall of each of the 16 operating conditions are calculated in this paper. Both precision and recall of every single category reach 100%. State-of-the-art baseline domain adaptation models (e.g., DANN, CORAL) achieve a maximum overall accuracy of only around 80%. Even for some baselines with global accuracy close to 90%, their confusion matrices still contain massive cross-misclassifications between different severity gradients within the same fault category and between the two opposite refrigerant imbalance faults. This result demonstrates that the hybrid attention mechanism can capture subtle time-series drifts, and the two-layer domain alignment strategy enables the features of identical faults to form compact clusters across different chillers. The proposed algorithm possesses practical engineering capability for hierarchical early warning, which intuitively highlights the superiority of the joint optimization framework integrating hybrid attention and multiple losses in distinguishing fine-grained fault boundaries.

## 6. Conclusions and Future Works

Aiming at the prominent problems of significant domain shift caused by heterogeneous equipment, scarce labeled samples in the target domain, interference from marine vibration noise, and difficulty in identifying multi-level subtle faults existing in marine screw chillers, this paper proposes a domain-adaptive fault diagnosis network named DAFDAN based on DDPN, together with a five-stage progressive training strategy and a joint multi-loss optimization scheme. The public centrifugal chiller dataset is adopted as the source domain, and measured data from self-developed marine screw chillers are used as the target domain for experimental verification. The core conclusions are summarized as follows:

(1) A dual-branch encoding structure consisting of a source domain compressor and a target domain extender is designed to adapt to the heterogeneous sensing dimensions of the two types of chillers. Residual connections are introduced to alleviate the vanishing gradient problem under few-shot training conditions. A hybrid attention module integrating SE-ECA channel attention and spatial attention is constructed to suppress noise and amplify time-series features of subtle faults. Ablation experiments verify that this module is critical to guarantee cross-domain recognition accuracy.
(2) A dual-layer domain alignment architecture combining explicit statistical constraints of MMD and implicit feature confusion via GRL-based adversarial training is established to reduce domain distribution discrepancies from multi-order statistics. Removing either of the two domain alignment losses leads to a sharp accuracy drop, and a single loss cannot eliminate coupled multi-dimensional domain shifts, which demonstrates their irreplaceable synergistic effect.
(3) A five-stage hierarchical training scheme is proposed, in which classification, reconstruction, adversarial, and KL semi-supervised losses are activated stage by stage with balanced weights to resolve gradient conflicts and training oscillations induced by multiple optimization objectives. Under identical network and loss configurations, the target-domain accuracy of single-stage end-to-end training only reaches 68.74%, far lower than the 99.98% achieved by the hierarchical training strategy, which proves that hierarchical training is an essential prerequisite for coordinated optimization of multiple losses.
(4) The KL semi-supervised loss makes full use of unlabeled target-domain samples to refine fault decision boundaries, while weighted cross-entropy mitigates sample imbalance. Comparative experiments reveal that DAFDAN outperforms baseline models, including SVM, DNN, MMD, CORAL, and DANN, by a large margin. The confusion matrix indicates zero misclassification and zero false alarms across all 16 operating conditions, with precision and recall rates of 100% for each fault category, enabling refined hierarchical early warning.
(5) Ablation, comparative, and visualization experiments collectively demonstrate that the hybrid attention module, multi-loss alignment mechanism, and five-stage training strategy work synergistically to address the few-shot cross-domain fault diagnosis challenges of marine chillers, providing algorithmic support for intelligent operation and maintenance of ship refrigeration equipment.

### 6.1. Limitations and Future Research Directions

Although the proposed model achieves a near-100% cross-domain diagnosis accuracy on laboratory-controlled simulation datasets, such results are obtained under fixed and simplified experimental conditions. There still exists a substantial gap between the current dataset and the actual offshore operating scenarios of marine vessels. Accordingly, this study has certain inherent limitations, and the marine chiller fault diagnosis task remains a challenging engineering problem requiring further in-depth exploration. In future work, we will conduct systematic research focusing on four practical and implementable directions: dataset expansion, compound fault identification, lightweight model deployment, and real-ship field validation. The detailed research motivations and technical implementation routes are elaborated as follows.

#### 6.1.1. Inherent Limitations of the Current Dataset

All time-series data of the target domain in this study are collected from a fixed indoor marine chiller simulation testbed, where the load adjustment range, vibration intensity, and ambient temperature and humidity are artificially controllable with single interference sources. In contrast, actual ship navigation involves multiple uncontrollable coupled disturbances, including continuous low-frequency hull vibration caused by varying sea conditions, drastic diurnal temperature fluctuations in the engine room, gradual pipeline fouling and sensor drift during long-term unit operation, and real-time dynamic load variations driven by ship speed and cabin cooling demands. The current dataset fails to cover such long-term degradation and multi-disturbance coupled operating conditions and only serves as a simplified verification platform, which cannot fully reproduce the complex feature distribution of real marine operating environments. Therefore, the high diagnosis accuracy achieved under laboratory conditions does not indicate that the cross-domain fault diagnosis task is intrinsically simple.

#### 6.1.2. Future Research Directions and Theoretical Basis

(1) Expansion of Multi-Source Real-Ship Fault Datasets

Research motivation: The current study only adopts data collected from a single laboratory screw chiller, which limits the coverage of equipment types, operating conditions, and fault modes, thereby restricting the generalization boundary of the proposed model. The lack of measured data from marine chillers with different tonnages and service years makes it difficult to validate the algorithm stability under multi-source and heterogeneous equipment scenarios.

Implementation scheme: We will cooperate with marine operation and maintenance laboratories and shipping enterprises to carry out field data collection. A portable multi-channel sensing acquisition terminal will be developed to continuously collect time-series data from marine chillers during offshore navigation. Long-term degradation fault samples, including pipeline fouling and sensor drift of aging units, will be synchronously acquired. A public multi-source marine refrigeration fault dataset with diverse equipment types, complex disturbances, and graded aging degrees will be constructed to compensate for the single-scene limitation of existing datasets.

(2) Identification of Multi-Coupled Compound Minor Faults

Research motivation: The current dataset only contains single-fault gradient conditions. However, two or more faults frequently occur simultaneously in practical marine maintenance scenarios, such as refrigerant leakage coupled with cooling water flow attenuation and pipeline fouling coupled with hull vibration. The time-series features of compound faults are mutually masked with highly ambiguous boundaries. The existing model is designed for single-fault recognition and lacks the capability of compound fault discrimination, which greatly hinders its practical engineering application.

Implementation scheme: Various compound fault conditions will be artificially constructed on the experimental platform to establish a multi-label sample library. Based on the existing DAFDAN architecture, a multi-branch multi-label classification head will be improved with hierarchical loss constraints. A novel domain-adaptive diagnosis algorithm targeting compound minor faults will be designed to address the feature confusion problem of coupled faults.

(3) Lightweight Model Development for Shipboard Edge Terminals

Research motivation: The complete DAFDAN network suffers from large parameter volume and high inference latency. Limited by the computing power and memory resources of embedded edge controllers in marine engine rooms, the original model cannot be directly deployed for real-time online fault warning. Model lightweighting is an essential prerequisite for the practical deployment of the algorithm on marine equipment.

Implementation scheme: A three-level compression strategy combining structured network pruning, 8-bit quantization, and teacher–student knowledge distillation will be adopted. The complete DAFDAN is treated as the teacher model to train a compact lightweight student network. The model parameters and single-sample inference time are compressed within a controllable accuracy loss range to adapt to the real-time diagnosis requirements of low-power shipboard edge devices.

(4) On-Site Deployment and Validation under Offshore Working Conditions

Research motivation: The simulated vibration and load disturbances in laboratory environments are essentially different from the continuous random vibration generated by offshore hull shaking. The real marine environment introduces numerous unknown noise offsets that cannot be fully reproduced in laboratory simulations. Models trained merely on bench datasets cannot be directly adapted to practical offshore scenarios.

Implementation scheme: The lightweight model will be transplanted to shipborne field acquisition hardware for long-term offshore field tests. The domain alignment module will be fine-tuned using real measured ship data to optimize the model generalization performance under strong random vibration and dynamic variable-load conditions, realizing the full-process verification of the algorithm from laboratory simulation to practical marine engineering deployment.

In summary, cross-domain fault diagnosis for heterogeneous marine chillers still faces multiple challenges, including single-scene dataset limitations, difficult compound fault identification, restricted edge deployment, insufficient real ship generalization capability, and intractable long-term temporal drift. This study only verifies the basic algorithm performance under simplified laboratory conditions. The above five research directions possess sufficient research potential and practical engineering value, which will be systematically explored in our future work.

## Figures and Tables

**Table 1:** Fault types of the source domain.

Fault Type	Normal State	Fault Severity	Abbreviation
Chilled water flow reduction	13.6 kg/s	10%, 20%, 30%	FWC
Cooling water flow reduction	17.0 kg/s	10%, 20%, 30%	FWE
Presence of non-condensable gas	0	100%, 1.70%, 2.40%	NC
Insufficient refrigerant	136 kg	10%, 20%, 30%	RL
Excessive refrigerant	136 kg	10%, 20%, 30%	RO

**Table 2:** Comprehensive Comparison of Discrepancies Between Source-Domain and Target-Domain Datasets.

Comparison Dimension	Source Domain (ASHRAE RP-1043 Centrifugal Chiller)	Target Domain (Laboratory Marine Screw Chiller)	Induced Domain Shift Effects
Core Chiller Equipment	Commercial chiller with centrifugal compressor	Marine-supported screw chiller	Differences in compressor dynamics inherently separate temperature-pressure time-series feature distributions of identical faults.
Number of sensor measuring points	28 monitoring measuring points	24 monitoring measuring points	Mismatched sensor dimensions and monitored physical quantities prevent shared feature extractors for the two datasets.
Rated operating conditions	Fixed rated load and steady-state commercial operating conditions	Dynamic variable load, simulated variable marine operating conditions	Continuous load fluctuations disturb data and shift feature mean/variance, widening inter-domain gaps.
Major fault categories	Five typical chiller faults	Five identical fault categories with different fault adjustment methods	Identical faults exhibit different parameter deviation ranges and change rates, leading to conditional distribution shifts.
Fault severity gradients	10%/20%/30% flow attenuation; refrigerant charge with fixed severity gradients	10%/20%/30% flow opening adjustment; graded charging of R134a and nitrogen	Inconsistent signal fluctuation amplitudes across fault severities hinder alignment of fine-grained fault boundaries.
Total sample size and labeling status	52,147 valid steady-state samples with complete labels	6782 valid steady-state samples, only 12% of samples with fault labels	Ample labeled source samples versus scarce labeled target samples result in an imbalanced label distribution shift.
Sources of data noise	Standard steady-state laboratory environment with minor interference	Complex industrial noise induced by vibration, frequent valve regulation, and load disturbance	Mismatched noise distributions obscure discriminative fault features and increase the difficulty of cross-domain alignment.

**Table 3:** Collected Variables of Target Domain Experimental Platform.

No.	Collected Variables	No.	Collected Variables
1	Compressor suction temperature	13	Chilled-water return temperature
2	Compressor discharge temperature	14	Heat-load inlet water temperature
3	Condenser inlet temperature	15	Heat-load outlet water temperature
4	Condenser outlet temperature	16	Chilled-water flow rate
5	Expansion-valve inlet temperature	17	Chilled-water inlet pressure
6	Expansion-valve outlet temperature	18	Chilled-water outlet pressure
7	Evaporator inlet temperature	19	Cooling-water inlet temperature
8	Evaporator outlet temperature	20	Cooling-water outlet temperature
9	Compressor discharge pressure	21	Cooling-water inlet pressure
10	Condenser inlet pressure	22	Cooling-water outlet pressure
11	Condenser outlet pressure	23	Compressor current
12	Expansion-valve inlet pressure	24	Compressor voltage

**Table 4:** Fixed Configuration of Loss Balance Weights for Each Stage.

Training Stage	$\lambda_{CE}$	$\lambda_{MMD}$	$\lambda_{MSE}$	$\lambda_{BCE}$	$\lambda_{KL}$
Stage 1 Source Domain Pre-training	1.0	0	0	0	0
Stage 2 Target Extender Warm-up	0	0	0.8	0	0
Stage 3 Adversarial Adaptation Training	1.0	0.3	0	0.6	0
Stage 4 Semi-supervised Training	1.0	0.3	0	0.6	0.2
Stage 5 Global Fine-tuning	1.0	0.3	0.1	0.6	0.2

**Table 5:** Metrics of Ablation Experiments on Core Network Modules.

Experimental Group	Source Accuracy	Source F1-Score	Target Accuracy	Target F1-Score	Std of Target Accuracy
Full DAFDAN	99.95%	99.94%	99.98%	99.97%	0.43%
Without SE-ECA + Spatial Hybrid Attention	92.15%	91.88%	77.93%	74.06%	4.89%
Remove Adversarial Training Module	99.39%	99.42%	68.74%	64.39%	3.82%
Remove Semi-supervised Learning Mechanism	93.07%	92.61%	76.15%	2.91%	2.91%

Note: The full DAFDAN group serves as the unified baseline model. This batch of module ablation experiments and the subsequent loss ablation experiments share the same dataset and training hyperparameters, resulting in identical baseline metric values.

**Table 6:** Quantitative Target-Domain Diagnosis Metrics under Different Loss Combinations.

Loss Combination Configuration	Target Accuracy	Target F1-Score	Standard Deviation of Accuracy
Full DAFDAN (Multi-loss + Five-stage Training + Hybrid Attention)	99.98%	99.97%	0.43%
Remove MMD Distribution Alignment Loss	62.41%	59.13%	4.16%
Remove BCE Adversarial Discriminant Loss	68.74%	64.39%	3.80%
Remove MSE Target Reconstruction Loss	83.52%	80.20%	2.05%
Remove KL Semi-supervised Consistency Loss	76.15%	70.77%	2.91%
Only Classification Loss (without any domain adaptation constraints)	77.93%	74.06%	4.89%
Only Single MMD Distribution Loss	31.66%	28.42%	5.77%
Only Single BCE Adversarial Loss	79.25%	76.01%	3.14%

Note: The full DAFDAN group serves as the unified baseline model. This set of loss ablation experiments shares the identical dataset and training hyperparameters with the previous module ablation experiments; thus, the baseline metric values remain consistent.

**Table 7:** Quantitative Target-domain Diagnosis Metrics under Different Training Strategies.

Training Method	Target Accuracy	Target F1-Score	Standard Deviation of Accuracy
Proposed Five-stage Progressive Training	99.98%	99.97%	0.43%
Conventional Single-stage End-to-end Synchronous Training	68.74%	64.39%	3.86%

## Data Availability

The original contributions presented in this study are included in the article. Further inquiries can be directed to the corresponding author.

## References

[1] Liu Y., Liang T., Zhang M., Jing N., Xia Y., Ding Q. (2024). Fault Diagnosis of Centrifugal Chiller Based on Extreme Gradient Boosting. Buildings.

[2] Bezyan Y., Nasiri F., Nik-Bakht M. (2026). Feature Selection and Fault Detection Under Dynamic Conditions of Chiller Systems. Electronics.

[3] Sun B., Liang D., Zhang H. (2024). An Improved Graph Deviation Network for Chiller Fault Diagnosis by Integrating the Sparse Cointegration Analysis and the Convolutional Block Attention Mechanism. Energies.

[4] Wang Z., Guo J., Zhou S., Xia P. (2023). Performance Evaluation of Chiller Fault Detection and Diagnosis Using Only Field-Installed Sensors. Sensors.

[5] Feng Q., Liu Y., Li Y., Chang G., Liang X., Su Y., Cao G. (2025). Study on a Fault Diagnosis Method for Heterogeneous Chiller Units Based on Transfer Learning. Entropy.

[6] Dou H., Zmeureanu R. (2023). Transfer Learning Prediction Performance of Chillers for Neural Network Models. Energies.

[7] Liu Y., Chen L., Shangguan D., Yu C. (2025). A Data-Driven Fault Diagnosis Method for Marine Steam Turbine Condensate System Based on Deep Transfer Learning. Machines.

[8] Cao G., Fu G., Feng Q., Ni M. (2024). Attention-Guided Domain Adaptation for Cross-Equipment Chiller Fault Diagnosis. Sensors.

[9] Nie L., Wu R., Ren Y., Tan M. (2023). Research on Fault Diagnosis of HVAC Systems Based on the ReliefF-RFECV-SVM Combined Model. Actuators.

[10] Zhang W., Li J., Huang S., Wu Q., Liu S., Li B. (2023). Application of Multi-Scale Convolutional Neural Networks and Extreme Learning Machines in Mechanical Fault Diagnosis. Machines.

[11] Wang X., Liu F., Zhao D. (2020). Cross-Machine Fault Diagnosis with Semi-Supervised Discriminative Adversarial Domain Adaptation. Sensors.

[12] Jin S., Jang A., Lee D., Kim S., Shin M., Do S. (2024). Development of Virtual Sensor Based on LSTM-Autoencoder to Detect Faults in Supply Chilled Water Temperature Sensor. Appl. Sci..

[13] Xiao Z., Yu L., Zhang H., Zhang X., Su Y. (2023). HVAC Load Forecasting Based on the CEEMDAN-Conv1D-BiLSTM-AM Model. Electronics.

[14] Xue Y., Chen H., Wang Y., Liu Y. (2026). A Fault Identification Method for EHA Multivariate Time Series Based on Multi-View Heterogeneous Ensemble Learning. Machines.

[15] Zhang P., Lin Y., Cui H., Gu J. (2024). Channel Attention-Based Conditional Diffusion Model Applied to Fault Diagnosis Under Imbalanced Data. Electronics.

[16] Lin H., Ho T., Tu C., Lin H., Yu C. (2025). MeTa Learning-Based Optimization of Unsupervised Domain Adaptation Deep Networks. Electronics.

[17] Guo Y., Zhang J., Sun B., Wang Y. (2023). Adversarial Deep Transfer Learning in Fault Diagnosis: Progress, Challenges, and Future Prospects. Sensors.

[18] Zou Y., Zhao W., Liu T., Zhang X., Shi Y. (2024). Research on High-Speed Train Bearing Fault Diagnosis Method Based on Domain-Adversarial Transfer Learning. Appl. Sci..

[19] Han H., Gao X., Han H., Gao H., Qi Y., Jiang K. (2025). Self-Supervised Learning via Domain Adaptive Adversarial Clustering for Cross-Domain Chiller Fault Diagnosis. IEEE Trans. Instrum. Meas..

[20] He K., Zhang X., Ren S., Sun J. (2016). Deep Residual Learning for Image Recognition. 2016 IEEE Conference on Computer Vision and Pattern Recognition (CVPR).

[21] Zhu Y., Chen W., Yan S., Zhang J., Zhu C., Wang F., Chen Q. (2025). Multi-Scale Residual Convolutional Neural Network with Hybrid Attention for Bearing Fault Detection. Machines.

[22] (1999). Fault Detection and Diagnostic (FDD) Requirements and Evaluation Tools for Chillers.

